# Predictors of telehealth use after the Minnesota Telehealth Act: analysis using the Minnesota All Payer Claims Database

**DOI:** 10.1093/haschl/qxae100

**Published:** 2024-08-16

**Authors:** Arkadipta Ghosh, Ethan Jacobs, Elizabeth Greener, Alyssa Evans, Mark Lee, Rui Wang, Pamela Mink, Michael Burian

**Affiliations:** Health Unit, Mathematica, Princeton, NJ 08540, United States; Health Unit, Mathematica, Cambridge, MA 02139, United States; Health Unit, Mathematica, Princeton, NJ 08540, United States; Health Unit, Mathematica, Princeton, NJ 08540, United States; Health Unit, Mathematica, Princeton, NJ 08540, United States; Health Unit, Mathematica, Princeton, NJ 08540, United States; Health Economics Program, Minnesota Department of Health, St. Paul, MN 55164, United States; Health Economics Program, Minnesota Department of Health, St. Paul, MN 55164, United States

**Keywords:** telehealth, ambulatory care visits, access to care, all-payer claims data

## Abstract

During the COVID-19 pandemic, the federal government and many state governments instituted expanded coverage for telehealth (TH) services and since have maintained it. Using data from the Minnesota All Payer Claims Database and publicly available data sources, we examined TH use among commercially insured and Medicare Advantage (MA) patients in Minnesota. In 2022, 30.4% of commercially insured patients and 24.4% of MA patients used TH services. Living in a metropolitan area, an area with a high proportion of Black, Indigenous, and People of Color residents, having greater disease burden, and being younger were associated with a greater likelihood of using TH. Living in an area with limited broadband access reduced the likelihood of TH use. Two patient subgroups more likely to use TH—younger patients in metropolitan areas and high-risk patients with depression—received a similar proportion of ambulatory visits via TH.

## Introduction

Telehealth use increased markedly during the COVID-19 pandemic and continued at relatively high levels as the pandemic waned.^1,2^ Responding to the pandemic, the federal government relaxed rules for reimbursing telehealth visits for Medicare patients, and several states initiated similar action. The Consolidated Appropriations Act of 2023 extended many of these Medicare provisions through December 31, 2024.^3^

The Minnesota Telehealth Act of 2021 expanded coverage for telehealth codifying changes that were temporarily instituted in March 2020 through executive orders. The act, which applies to commercial health insurance and Minnesota Health Care Programs (including Medicaid), prevents limits on coverage for telehealth services based on geography, increases the accessibility of behavioral health and substance use disorder services, adds audio-only visits to the definition of telehealth, and extends payment parity by requiring equivalent reimbursement for audio-only visits as for in-person services.

The act directed the Minnesota Department of Health to study the impact of telehealth expansion and payment parity, focusing on private insurance and examining disparities in telehealth use and health outcomes.^4^ Submitted to the Minnesota Legislature in May 2023, the preliminary report found that patients and providers valued telehealth for its flexibility and convenience, telehealth expanded access to behavioral health and specialist services, and rural patients used audio-only telehealth to access care given challenges in using audiovisual telehealth.^5^

The provisions in the act are consistent with federal policies. Some federal and state provisions will, however, sunset without further action. For example, the requirement to reimburse audio-only telehealth at parity with in-person care expires in Minnesota on July 1, 2025. The Centers for Medicare & Medicaid Services will not cover audio-only services, except for mental health, after December 31, 2024. Other states are also determining which telehealth policies to continue.

Decisions on ending telehealth flexibilities should be informed by the potential impact on patients, particularly those facing barriers to accessing in-person care or audiovisual telehealth. Telehealth expansion during COVID-19 likely improved access for many patients with preexisting barriers. Although state-level telehealth policies were associated with increased availability of mental health services,^6^ it is unclear whether ending the greater telehealth flexibilities will adversely affect health outcomes, equity, and health care spending.

Critical to answering these questions is understanding which patients are more likely to use telehealth and what drives the use when telehealth access is expanded. Analyzing the 2022 Health Information National Trends Survey, one recent study found that patients with higher health needs were more likely to use telehealth services; also, older patients, those with insufficient internet access, and uninsured patients were more likely to use audio-only telehealth.^7^ Other studies relying on survey data from 2021 onward found higher self-reported telehealth use among women, college graduates, Hispanic patients, adults under age 65, and patients with multiple chronic conditions or depression; telehealth use was lower among rural patients and uninsured patients, with mixed results for Black patients.^8-13^ Other studies conducted during the pandemic found that patients older than 65, those who are Black, Indigenous, and People of Color (BIPOC), dually eligible for Medicare and Medicaid, in rural areas, and in neighborhoods with low socioeconomic status (SES) or low internet access were more likely to rely on audio-only telehealth.^14-16^ A study based on a survey of physicians identified limited access to technology, limited digital literacy, and lack of broadband access as significant barriers to telehealth use.^17^ An analysis of the American Life Panel Omnibus Survey found that younger adults (20-39 years) and Hispanic patients preferred video visits, while Black patients preferred in-person care.^18^ Findings from the Minnesota Health Access Survey show the racial gap in insurance coverage widened in 2021,^19^ potentially lowering access to telehealth services among BIPOC Minnesotans.

We extend the analysis described in the preliminary report to the Minnesota Legislature for the Minnesota Telehealth Study. We used data from the Minnesota All Payer Claims Database (MN APCD) to examine predictors of telehealth use in 2022—when health care use returned to pre-pandemic levels—among commercially insured and Medicare Advantage (MA) patients. By focusing on telehealth users or what predicted telehealth use in a period following the implementation of the Minnesota Telehealth Act and after the worst phase of the pandemic, we sought to provide policymakers with critical information on patient subgroups more likely to be affected by future telehealth policies. Also, for patient subgroups predicted to have a higher likelihood of telehealth use, we examined ambulatory care use to better understand telehealth use patterns, for instance, whether the proportion of telehealth visits differed based on potential underlying factors.

Although states differ in their telehealth policies, many of the patterns observed in this study could apply to other states. Also, policymakers beyond those in Minnesota are likely to be interested in understanding the potential impact of withdrawing flexibilities with audio-only visits. Along with commercially insured patients, we include MA patients in our analysis since MA plans are administered by commercial insurers and including the MA population allows us to examine findings for elderly patients. There is limited research on telehealth utilization among MA patients and even fewer studies using data from a state all-payer claims database to examine telehealth use among commercially insured patients. Our study, therefore, fills critical gaps in the literature and goes beyond self-reports in survey data to assess telehealth use from claims.

## Data and methods

### Data sources

We used data from the MN APCD, Extract 26, specifically enrollment data and medical claims for Minnesotans in commercial or MA plans in 2021 and 2022. The MN APCD has been used in prior research to examine the use of telehealth services.^20^ It includes virtually all (over 95%) MA members in Minnesota, and about 40% of commercial members, most of whom are enrolled in fully insured employer-based plans or individual market plans. We excluded Medicare Cost enrollees from this analysis (Appendix A describes the MN APCD).

Using rural–urban commuting area codes derived from the ZIP Code-level member data in the MN APCD, we assigned patients to metropolitan or nonmetropolitan areas. We used the Johns Hopkins Adjusted Clinical Group (ACG) System version 13.0 output incorporated in the MN APCD to assign chronic condition markers and patient risk scores.

Supplemental data sources included the National Plan and Provider Enumeration System and Provider Enrollment, Chain, and Ownership System data on provider specialties, county-level data on quarterly COVID-19 hospitalization rates in 2021 from MDH, and ZIP Code-level data from the American Community Survey on the percentage of households with broadband access, living in poverty, or BIPOC (see Appendix A).

### Measures of telehealth use

We identified claims with relevant place-of-service codes, procedure codes, or procedure code modifiers as telehealth, excluding claims for telehealth facility fees and provider-to-provider communication.

We assigned each telehealth visit to one of three subcategories, consistent with the Minnesota Telehealth Act's definition of telehealth visits (see Table B.1 in Appendix B for details):

- Audiovisual telehealth that involves telecommunications technology with both audio and visual components.
- Audio-only telehealth that involves telecommunications technology with only an audio connection (eg, a telephone visit).
- Other telehealth such as e-visits through patient portals and asynchronous store-and-forward telehealth services.

We examined two outcomes: (1) use of any of the three types of telehealth visits and (2) use of audio-only telehealth (which may be the only form of telehealth accessible to patients with no broadband access or with low technological literacy). We also examined the proportion of primary care, specialist, and behavioral health visits delivered via telehealth (see Appendix C for definitions of ambulatory care visits).

### Analyses

All analyses were conducted separately for commercially insured patients and MA patients. To identify the key predictors of telehealth use, we estimated odds ratios (ORs) from separate logistic regressions predicting any telehealth use and audio-only telehealth use in 2022 as a function of patient and area characteristics in 2021 (see Appendix D). To distinguish between significant predictors of telehealth use for the elderly vs nonelderly patients, we restricted the commercial and MA samples to patients under 65 years and those 65 or older, respectively, excluding 3%-4% of patients (Table D.2 in Appendix D).

Regressions included indicators for the following patient characteristics: age categories, gender, high-risk status (whether above the 75th percentile of the Johns Hopkins ACG risk score distribution), and ACG condition markers for specific chronic and behavioral health conditions diagnosed in at least 10% of the commercial and MA populations (depression, diabetes, hypertension, lipid metabolism disorders, glaucoma, hypothyroidism, ischemic heart disease, low back pain, and persistent asthma). Indicators for area-level characteristics based on ZIP Codes included metropolitan status, lowest decile of the percentage of households with broadband access vs upper deciles, highest quartile of the percentage of BIPOC residents vs lower quartiles, highest quartile of the percentage of households in poverty vs lower quartiles, and quarterly county-level COVID-19 hospitalization rates in 2021 (Table D.1 in Appendix D lists explanatory variables and data sources). We did not impute missing values for any explanatory variable and excluded 2% and 9% of the MA and commercial patient samples, respectively, with missing information (see Appendix E).

Based on the results from the logistic regressions, we examined ambulatory care use among patient subgroups more likely to use telehealth and whose telehealth use was either likely to be driven by considerations of convenience and access or by high health care needs. This helped us examine whether the extent of telehealth use differed based on underlying factors and whether telehealth was likely to substitute for or supplement in-person visits.

### Limitations

Claims may have incomplete or inaccurate diagnosis or procedure codes either because some diagnoses are not captured or individual providers vary in their coding practices. Because our analysis excluded claims that were denied or not covered by insurance, our results may undercount telehealth or in-person visits to behavioral health providers who may not accept insurance.

Audio-only telehealth visits may be undercounted in claims, as shown in prior research.^21^ Providers often use the same CPT codes for billing both audio-only visits and audiovisual telehealth visits, especially since a visit scheduled as an audiovisual visit could be delivered as audio only due to technological challenges.

Because the MN APCD's commercial claims exclude most self-insured patients, our findings pertain mainly to fully insured commercial members. Since the MN APCD does not include patient-level data on race and ethnicity, we rely on area-level data to construct proxies. Therefore, potential bias from unmeasured patient characteristics is a concern. For instance, patients’ SES is likely an important determinant of health and health care use, including telehealth visits. Although we proxy for patient SES using area characteristics such as poverty and race/ethnicity mix, unobserved patient SES could bias the estimated relationship between other patient characteristics like chronic conditions and telehealth use. Therefore, we do not attribute causality to any of our findings.

## Results

### Telehealth use in 2022

**Figure 1.**
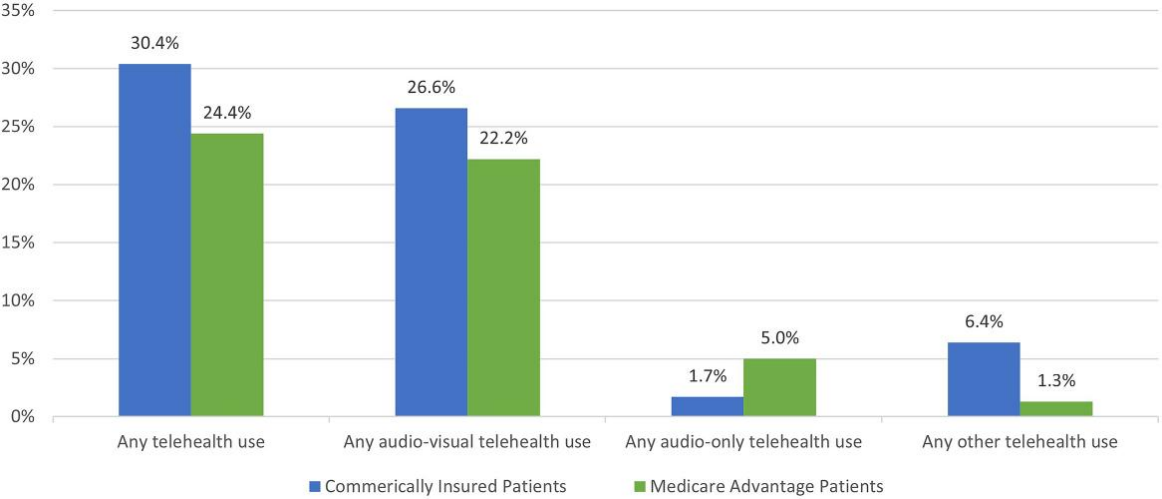
Percentages of telehealth users among commercially insured and MA patients in Minnesota in 2022. Source: Authors’ analysis of claims data from the MN APCD, Extract 26. Notes: The 3 types of telehealth use are not mutually exclusive categories. Therefore, the sum of the percentages of users for the 3 types of telehealth could exceed the percentage of users for any telehealth. Other telehealth includes e-visits, virtual check-ins, online/digital assessments, and remote evaluation of recorded video and/or images submitted by a patient. Table B.1 in Appendix B provides additional details on the definition of different telehealth visits.

**Figure 2.**
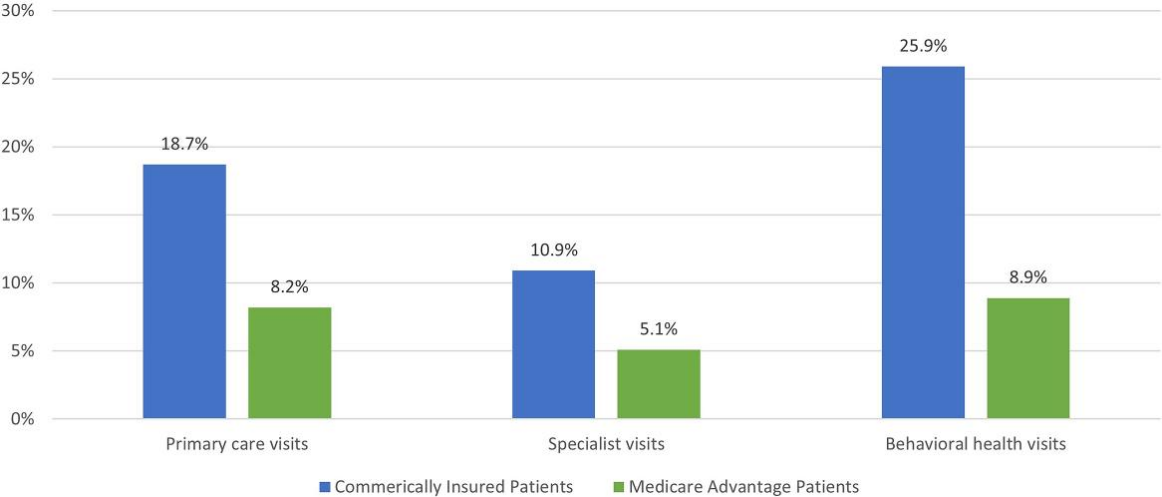
Percentage of ambulatory care visits done via telehealth among commercially insured and MA patients in Minnesota in 2022. Source: Authors’ analysis of claims data from the MN APCD, Extract 26. Notes: Provider specialties are based on cross walking the claims data to the National Plan and Provider Enumeration System and the Provider Enrollment, Chain, and Ownership System. Tables C.1-C.3 in Appendix C provides additional details on the identification of ambulatory care visits by type of visit.

The study sample included 874 251 commercially insured patients and 346 010 MA patients who were enrolled in a commercial or MA plan for at least 3 months in both 2021 and 2022, had at least 1 medical claim in both years, and had a valid Minnesota ZIP Code (Tables E.1 and E.2 in Appendix E). Commercially insured patients had slightly higher rates of telehealth use than MA patients: 30.4% vs 24.4%, respectively, used any telehealth (Figure 1). Audiovisual telehealth visits constituted the most telehealth use for both groups: 26.6% of commercially insured patients and 22.2% of MA patients used audiovisual telehealth services. The use of audio-only telehealth services, though limited, was more common among MA patients (5%) than among commercially insured patients (2%). Among both groups, telehealth represented a higher share of behavioral health visits compared with primary care and specialist visits: 25.9% and 8.9% of behavioral health visits for commercially insured and MA patients, respectively, were delivered through telehealth (Figure 2).

### Drivers of telehealth use

#### Commercially insured patients

We identified several significant predictors of telehealth use among commercially insured patients under age 65 (Table 1). Living in a metropolitan area (OR: 2.12; 95% CI, 1.94-2.33; *P* < 0.001) and in ZIP Codes with a relatively high percentage of BIPOC residents (OR: 1.21; 95% CI, 1.11-1.31; *P* < 0.001) was associated with significantly higher odds of using telehealth. Patients in ZIP Codes in the lowest decile of broadband access vs all upper deciles combined had lower odds of using any telehealth (OR: 0.82; 95% CI, 0.74-0.92; *P* < 0.01). Among patient characteristics, being high risk (OR: 1.89; 95% CI, 1.86-1.92; *P* < 0.001), having depression (OR: 2.41; 95% CI, 2.37-2.46; *P* < 0.001) and being age 18-44 (OR: 1.66; 95% CI, 1.64-1.69; *P* < 0.001) were associated with higher odds of using telehealth, as were the presence of most chronic conditions. Results for audio-only telehealth use were similar but with statistically nonsignificant ORs on area characteristics.

**Table 1.** ORs and CIs estimated from logistic regressions predicting any use of telehealth and any use of audio-only telehealth among commercially insured and MA patients in Minnesota in 2022.

	Commercially insured patients under age 65		Medicare Advantage patients age 65 or older	
Patient characteristics	Any telehealth use	Any audio-only telehealth use	Any telehealth use	Any audio-only telehealth use
Commercially insured patient is 45-64 (reference category)			NA	NA
Commercially insured patient is under 18	1.00 (0.98-1.02)	0.56*** (0.48-0.64)	NA	NA
Commercially insured patient is 18-44	1.66*** (1.64-1.69)	1.08** (1.02-1.13)	NA	NA
MA patient is 65-74 (reference category)	NA	NA		
MA patient is 75-84	NA	NA	0.86*** (0.84-0.87)	1.07** (1.03-1.11)
MA patient is 85+	NA	NA	0.72*** (0.70-0.75)	0.88*** (0.83-0.94)
Patient is male (vs female)	0.72*** (0.71-0.73)	0.90*** (0.87-0.93)	0.90*** (0.89-0.92)	0.94** (0.90-0.98)
Patient is high-risk (vs not high-risk)	1.89*** (1.86-1.92)	1.78*** (1.69-1.87)	1.71*** (1.67-1.75)	1.86*** (1.77-1.96)
Whether patient has vs does not have a specific chronic condition:				
Depression	2.41*** (2.37-2.46)	1.55*** (1.47-1.62)	1.66*** (1.63-1.7)	1.39*** (1.34-1.44)
Diabetes	1.12*** (1.09-1.15)	1.30*** (1.21-1.39)	1.01 (0.99-1.03)	1.02 (0.98-1.06)
Hypertension	1.10*** (1.08-1.12)	1.17*** (1.11-1.23)	1.06*** (1.04-1.09)	1.13*** (1.08-1.17)
Lipid metabolism disorder	0.86*** (0.84-0.87)	0.97 (0.91-1.03)	1.00 (0.98-1.02)	0.97 (0.93-1.01)
Glaucoma	0.93** (0.90-0.97)	1.06 (0.95-1.19)	1.01 (0.98-1.03)	1.04 (0.99-1.09)
Hypothyroidism	1.14*** (1.12-1.16)	1.12** (1.05-1.19)	1.12*** (1.10-1.15)	1.07** (1.03-1.12)
Ischemic heart disease	1.06** (1.02-1.11)	1.51*** (1.37-1.67)	1.13*** (1.10-1.16)	1.20*** (1.15-1.26)
Lower back pain	1.15*** (1.13-1.17)	1.26*** (1.20-1.32)	1.26*** (1.23-1.29)	1.25*** (1.21-1.3)
Persistent asthma	1.38*** (1.35-1.40)	1.36*** (1.29-1.43)	1.29*** (1.26-1.33)	1.24*** (1.18-1.3)
Community characteristics				
Lowest decile of broadband access (vs upper deciles)	0.82** (0.74-0.92)	1.34 (0.9-1.99)	0.78*** (0.68-0.88)	0.90 (0.70-1.14)
Highest quartile of the percentage of residents in poverty (vs lower quartiles)	0.98 (0.90-1.07)	1.18 (0.98-1.41)	0.95 (0.85-1.06)	1.06 (0.93-1.21)
Highest quartile of the percentage of BIPOC residents (vs lower quartiles)	1.21*** (1.11-1.31)	0.96 (0.86-1.07)	1.21*** (1.11-1.33)	1.09 (0.99-1.21)
In a metropolitan area (vs nonmetropolitan area)	2.12*** (1.94-2.33)	0.70 (0.46-1.09)	1.86*** (1.65-2.08)	0.99 (0.68-1.44)
COVID-19 hospitalizations in Q1 of 2021	1.005*** (1.004-1.007)	0.999 (0.996-1.002)	1.004*** (1.002-1.005)	1.000 (0.997-1.002)
COVID-19 hospitalizations in Q2 of 2021	1.005*** (1.003-1.006)	1.003 (0.998-1.008)	1.005*** (1.003-1.007)	1.000 (0.995-1.005)
COVID-19 hospitalizations in Q3 of 2021	1.000 (0.999-1.002)	0.999 (0.997-1.002)	1.00 (0.998-1.001)	0.999 (0.997-1.001)
COVID-19 hospitalizations in Q4 of 2021	0.997*** (0.996-0.998)	0.998 (0.995-1.001)	0.997*** (0.996-0.998)	1.000 (0.997-1.003)
Constant	0.10*** (0.08-0.12)	0.02*** (0.01-0.04)	0.12*** (0.09-0.15)	0.04*** (0.02-0.07)
Number of observations	848 515	848 515	331 737	331 737
Adjusted *R*^2^	0.0953	0.0447	0.0581	0.0255

Source: Authors’ analysis of claims data from the MN APCD, Extract 26; rural–urban commuting area codes from the US Department of Agriculture; risk scores and condition markers from version 13.0 of the Johns Hopkins ACG System output; provider specialties from the National Plan and Provider Enumeration System and the Provider Enrollment, Chain, and Ownership System; and area-level population characteristics from the American Community Survey.

Notes: We restricted the regression samples to commercial and MA patients under 65 years and those 65 or older, respectively, excluding 3% of commercially insured patients and 4% of MA patients. This resulted in sample sizes that were slightly smaller than the 874 251 commercially insured and 346 010 MA patients included in the analysis of telehealth use in 2022. Patients with risk scores above the 75th percentile of the Johns Hopkins ACG risk score distribution were classified as high-risk patients.

Abbreviations: BIPOC, Black, Indigenous, and People of Color; MA, Medicare Advantage.

^**^ *P* < 0.01.

***P < 0.001.

#### MA patients

Among MA patients ages 65 and older, significant predictors of telehealth use were in line with those for commercially insured patients under 65 (Table 1), with the highest ORs for residing in a metropolitan area (OR: 1.86; 95% CI, 1.65-2.08; *P* < 0.001), being high risk (OR: 1.71; 95% CI, 1.67-1.75; *P* < 0.001), and having depression (OR: 1.66; 95% CI, 1.63-1.70; *P* < 0.001). Patients 75-84 (OR: 0.86; 95% CI, 0.84-0.87; *P* < 0.001) and those 85+ (OR: 0.72; 95% CI, 0.70-0.75; *P* < 0.001) were less likely to use telehealth. These findings were consistent for the use of audio-only telehealth, although none of the area characteristics were significant predictors.

### Patterns of ambulatory care use among patients more likely to use telehealth

Based on patient and area characteristics with the highest ORs for telehealth use, we examined ambulatory care use and patterns of telehealth use among specific patient subgroups. We focused on two subgroups whose telehealth use was either likely to be driven by considerations of convenience and access or by high health care needs. Among commercially insured patients under 65, these two subgroups were (1) patients 18-44 years old living in metropolitan areas, who are likely to have better access to in-person visits (eg, due to shorter travel times) as well as to more reliable broadband internet and cellular services for telehealth, and (2) patients who were high risk and had depression, regardless of their location, whose health care use, including use of telehealth, was more likely to be driven by disease burden. Similarly, among MA patients 65 and older, the two subgroups we examined were (1) patients aged 65-74 living in metropolitan areas and (2) patients who were high risk and had depression. These subgroups are not mutually exclusive, and our goal in comparing these subgroups was to examine whether patterns of telehealth use differed by underlying need or motivation for telehealth use.

Among commercially insured patients under 65, we found similar rates of telehealth use among patients aged 18-44 residing in metropolitan areas and those who were high risk and had depression, although as expected, patients in the latter group had a higher volume of total ambulatory (in-person and telehealth) visits (Table 2). On average, patients aged 18-44 in metropolitan areas had 1.7 primary care provider (PCP) visits, 5 behavioral health (BH) visits, and 0.9 specialist visits in 2022. Around 25% of PCP and BH visits and 12% of specialist visits were via telehealth, with the proportion of audio-only telehealth visits being 0.5% or less. Although high-risk patients with depression had more visits—3.3 PCP visits, 11 BH visits, and 2.2 specialist visits—the proportion of telehealth visits was not higher than for patients in the other group. Specifically, around 25% of PCP and BH visits and 15% of specialist visits were via telehealth. The proportion of audio-only telehealth visits was 0.7% or less.

**Table 2.** Number of ambulatory care visits and percentage of visits via telehealth among patient subgroups more likely to use telehealth.

	Commercially insured, 18-44, metropolitan (*N* = 269 964; average risk score = 0.54)			Commercially insured, high-risk, with depression (*N* = 86 837; average risk score = 1.74)		
	PCP visits	BH visits	Specialist visits	PCP visits	BH visits	Specialist visits
Annualized number of visits						
Mean	1.72	4.98	0.92	3.29	10.91	2.18
SD	2.31	11.05	1.68	3.47	18.76	3.00
Percentage of TH visits	24.13%	24.64%	12.00%	23.28%	26.44%	15.40%
Percentage of audio-only TH visits	0.49%	0.34%	0.33%	0.73%	0.57%	0.49%

	Medicare Advantage, 65-74, metropolitan (*N* = 132 579; average risk score = 1.38)			Medicare Advantage, high-risk, with depression (*N* = 27 805; average risk score = 3.58)		
	PCP visits	BH visits	Specialist visits	PCP visits	BH visits	Specialist visits
Annualized number of visits						
Mean	2.80	4.89	2.33	4.51	8.35	3.46
SD	2.93	6.34	2.66	4.47	9.56	3.62
Percentage of TH visits	8.43%	7.27%	4.31%	9.39%	9.91%	6.87%
Percentage of audio-only TH visits	0.84%	0.70%	0.56%	1.42%	1.30%	1.18%

Source: Authors’ analysis of claims data from the MN APCD, Extract 26 and data on Rural-Urban Commuting Area codes from the US Department of Agriculture, risk scores and condition markers from version 13.0 of the Johns Hopkins ACG System output, provider specialties from the National Plan and Provider Enumeration System and the Provider Enrollment, Chain, and Ownership System, and area-level characteristics from the American Community Survey.

Notes: We identified the commercially insured and MA patient subgroups more likely to use telehealth based on patient and area characteristics with the highest ORs in the logistic regressions predicting any use of telehealth and any use of audio-only telehealth.

Abbreviations: BH, behavioral health; PCP, primary care provider; TH, telehealth.

The pattern of telehealth use was similarly consistent across the two MA patient subgroups (Table 2). For PCP, BH, and specialist visits, these two groups received at most 10% of visits via telehealth. High-risk MA patients with depression had somewhat higher rates of audio-only telehealth use compared with those in metropolitan areas (up to 1.4% of visits vs 0.8% or less).

## Discussion

Our findings show substantial telehealth use in 2022 among commercially insured and MA patients in Minnesota, following the 2021 Minnesota Telehealth Act. High-risk patients, patients diagnosed with depression, relatively younger patients, and those in metropolitan areas were more likely to use telehealth than other patients.

However, even among high telehealth users—high-risk patients with depression and younger patients in metropolitan areas—telehealth accounted for at most a quarter of ambulatory care visits. This finding is consistent with national survey data showing that while most patients prefer to receive some of their services via video visits, they also prefer in-person services when given the choice.^17^

Currently, MA patients rely on in-person visits for most of their care. Even among MA patient subgroups with greater likelihood of telehealth use, fewer than 10% of primary care and behavioral health visits, and fewer than 7% of specialist visits, were delivered via telehealth. This pattern is consistent with more complex health care needs, preference for conventional in-person visits, and lower technological literacy among elderly patients. Commercially insured patients with high telehealth use obtained a quarter of primary care and behavioral health visits and up to 15% of specialist visits via telehealth, consistent with a greater reliance on and comfort with using technology for health care, a preference for the convenience of telehealth, and less complex medical and behavioral health issues.

Comparing two groups of patients more likely to use telehealth—high-risk patients with depression and younger patients in metropolitan areas—we found that the proportion of ambulatory visits conducted via telehealth was similar, despite the greater disease burden and higher volume of visits in the first group. Convenience and widespread access to broadband internet are as important as higher health care need in driving the use of telehealth services. If ease of access drives some telehealth use and telehealth is not a perfect substitute for in-person visits, the total number of ambulatory visits is likely to increase with expanded access to telehealth.^22^

Although the use of audio-only visits was low with possible underreporting in claims, some patients relied on it across urban and rural areas of Minnesota. Also, MA patients were more likely to use audio-only visits than commercially insured patients, especially high-risk MA patients with depression. Among elderly patients with a high disease burden, potential mobility issues, and challenges with using audiovisual communication technology, the ability to consult a provider via telephone could be a valuable option for accessing care.

## Conclusion

In the absence of policies supporting greater audiovisual telehealth use or promoting access to in-person care, limiting access to audio-only telehealth could adversely affect high-need patients more than others. Further research is needed to understand how telehealth use affects outcomes among high-need patients and whether expanded access to telehealth comes at the expense of or enhances patient satisfaction and care quality.

## Supplementary Material

qxae100_Supplementary_Data
