# Supplementary material for: Predictors of telehealth use after the Minnesota Telehealth Act: analysis using the Minnesota All Payer Claims Database
Source: Health Aff Sch. 2024 Aug 16;2(8):qxae100. doi: 10.1093/haschl/qxae100 (PMC11350286; doi:10.1093/haschl/qxae100)
Supplement: qxae100_Supplementary_Data [file qxae100_supplementary_data.zip › HAS_telehealth_appendices_Revised_clean.docx]

PREDICTORS OF TELEHEALTH USE AFTER THE MINNESOTA TELEHEALTH ACT: ANALYSIS USING THE MINNESOTA ALL PAYER CLAIMS DATABASE

Supplementary Appendices

Appendix A

Data Sources

The primary data source for our analysis is the Minnesota All Payer Claims Database (MN APCD), Extract 26. The MN APCD is a state repository of de-identified health care claims data that is derived from billing records sent by medical providers to insurance companies, plan administrators, and public payers and includes de-identified claims data for dates of service beginning on January 1, 2008. The MN APCD systematically collects and integrates medical claims, pharmacy claims, and eligibility files from both private and public payers and covers claims in all care settings, including inpatient hospitalizations, outpatient office visits, laboratory and imaging services, behavioral health services, durable medical equipment, telehealth, as well as pharmacy claims. Health plan companies with less than $3 million in annual medical claims and/or $300,000 in annual pharmacy claims are exempt from data submission requirements of the MN APCD. Additionally, self-insured group plans are not required to submit data to the MN APCD. Commercial enrollees in the MN APCD represent Minnesotans with individual or small group plans, including those available through the state exchange (MNSURE), as well as those covered by fully insured large group plans. The Minnesota Department of Health estimated that data on 40 percent of the commercially insured population were included in the MN APCD.^[[1]](#footnote-2)^ Although the incompleteness of claims data for commercially insured patients is a limitation, the commercial population in the MN APCD does include those to whom state policies would apply.

We used the enrollment and claims data from the MN APCD in 2021 and 2022 to define the study population, identify the use of telehealth and other services, and define outcomes of interest, focusing on commercially insured patients and Medicare Advantage (MA) beneficiaries. The MN APCD includes over 95% of Medicare beneficiaries, including those enrolled in MA plans. We excluded patients enrolled in Medicare Cost programs from the analysis on MA patients.

We also relied on several supplemental data sources for this study.

Supplemental data within the MN APCD include:

- Rural–Urban Commuting Area (RUCA) codes: Allowed us to assign patients to metropolitan and nonmetropolitan areas
- Version 13.0 of the Johns Hopkins Adjusted Clinical Group System output: Allowed us to stratify patients by chronic conditions and risk scores

Other supplemental data sources that we used are:

- National Plan and Provider Enumeration System (NPPES) and Provider Enrollment, Chain, and Ownership System (PECOS): Includes data on provider specialties
- County-level data on quarterly COVID-19 hospitalization rates in 2021 from the Minnesota Department of Health
- American Community Survey (ACS) data: Linking patient Zip Code data from the MN APCD to Zip Code-level data from the ACS allowed us to identify the percentage of the population who have broadband access, live in poverty, or are racial or ethnic minorities.

Appendix B

Identifying Telehealth Services

The place of service (POS)^[[2]](#footnote-3)^, modifier, and procedure codes used to identify telehealth services and to classify those services as audiovisual, audio only, or other are listed in Table B.1. We specifically assigned each telehealth visit to one of three categories, consistent with the Minnesota Telehealth Act’s definition of telehealth:

- Audiovisual telehealth (visits using telecommunications technology that involves both audio and visual components, such as an audiovisual visit conducted using a computer with internet connection)
- Audio-only telehealth (visits using telecommunication technology that involves an audio connection without any visual component, such as telephone Evaluation & Management [E&M] services)
- Other telehealth (such as e-visits through patient portals and asynchronous store-and-forward telehealth services)

Because this study focuses on the delivery of telehealth services to patients, we excluded claims for telehealth facility fees and provider-to-provider communication.

**Table B.1.** Codes used to identify claims for services delivered via telehealth.

| Code Type | Code | Description | Telehealth Type |
| --- | --- | --- | --- |
| POS | 02 | Use of telecommunication technology | Audio-visual |
| POS | 10 | Use of telecommunication technology | Audio-visual |
| Modifier | 93 | Synchronous telemedicine service rendered via telephone or other real-time interactive audio-only telecommunications system | Audio only |
| Modifier | FQ | Service was provided using audio-only communication technology | Audio only |
| Modifier | 95 | Synchronous telemedicine A/V | Audio-visual |
| Modifier | GT | Via telecommunications system e.g., video | Audio-visual |
| Modifier | G0 | Telehealth services for diagnosis, evaluation, or treatment of symptoms of an acute stroke | Audio-visual |
| Modifier | GQ | Use of asynchronous telecommunications system | Other |
| Procedure (CPT) | 99441 | Telephone E&M services | Audio only |
| Procedure (CPT) | 99442 | Telephone E&M services | Audio only |
| Procedure (CPT) | 99443 | Telephone E&M services | Audio only |
| Procedure (CPT) | 98966 | A nonphysician provider telephone E&M services, 5-10 mins | Audio only |
| Procedure (CPT) | 98967 | A nonphysician provider telephone E&M services, 11-20 mins | Audio only |
| Procedure (CPT) | 98968 | A nonphysician provider telephone E&M services, 21-30 mins | Audio only |
| Procedure (HCPCS) | G2552 | Brief communication technology-based service, e.g., virtual check-in, by a qualified health care professional | Audio only |
| Procedure (HCPCS) | G2552 | Brief communication technology-based service, e.g., virtual check-in, by a qualified health care professional | Audio only |
| Procedure (HCPCS) | G2025 | RHC/FQHC distant site telehealth service | Audio-visual |
| Procedure (CPT) | 98970 | Online digital assessment and management by nonphysicians – e-visits through patient portal | Other |
| Procedure (CPT) | 98971 | Online digital assessment and management by nonphysicians – e-visits through patient portal | Other |
| Procedure (CPT) | 98972 | Online digital assessment and management by nonphysicians – e-visits through patient portal | Other |
| Procedure (CPT) | 99421 | Online digital E&M by physicians and qualified professionals such as NPs – e-visits through patient portal | Other |
| Procedure (CPT) | 99422 | Online digital E&M by physicians and qualified professionals such as NPs – e-visits through patient portal | Other |
| Procedure (CPT) | 99423 | Online digital E&M by physicians and qualified professionals such as NPs – e-visits through patient portal | Other |
| Procedure (CPT) | 99444 | Brief check-in or e-visit | Other |
| Procedure (CPT) | 98969 | Brief check-in or e-visit | Other |
| Procedure (HCPCS) | G0071 | Communication technology-based services for 5 minutes or more of a virtual communication between a rural health clinic or federally qualified health center practitioner and patient | Other |
| Procedure (HCPCS) | G2012 | Brief communication technology-based service, e.g., virtual check-in, by a physician or other qualified health care professional who can report evaluation and management service | Other |
| Procedure (HCPCS) | G2061 | Online Assessment of established patient by Qualified Nonphysician Healthcare Professional, 5-10 mins | Other |
| Procedure (HCPCS) | G2062 | Online Assessment of established patient by Qualified Nonphysician Healthcare Professional, 11-20 mins | Other |
| Procedure (HCPCS) | G2063 | Online Assessment of established patient by Qualified Nonphysician Healthcare Professional, 21+ mins | Other |
| Procedure (HCPCS) | G2010 | Remote evaluation of recorded video and/or images submitted by an established patient (e.g., store and forward) | Other |
| Procedure (HCPCS) | G2250 | Remote assessment of recorded video and/or images submitted by an established patient (e.g., store and forward) | Other |
| Procedure (HCPCS) | G2251 | Brief communication technology-based service, e.g., virtual check-in, by a qualified health care professional | Other |
| Procedure (HCPCS) | G2252 | Brief communication technology-based service, e.g., virtual check-in, by a qualified health care professional | Other |

POS = Place of service; CPT = Current Procedural Terminology; E&M = Evaluation and management; HCPCS = Healthcare Common Procedure Coding System; NP = Nurse practitioner.

Appendix C

Classifying Ambulatory Care Services

The following methodology was used to classify claims as primary care, specialty care, or behavioral health visits:

*Primary care visits.* We classified an in-person or telehealth claim as a primary care claim if (1) the service was delivered by a primary care practitioner, including selected specialties of physicians, nurse practitioners, clinical nurse specialists, and physician assistants, and (2) it had a primary care procedure code with place of service (POS) restrictions^[[3]](#footnote-4)^. The provider taxonomy codes, procedure codes, and POS restrictions we used to identify primary care visits are listed in Table C.1 and Table C.2.

*Specialty care visits.* We classified an in-person or telehealth claim as a specialist claim if the service was delivered by a specialty provider, such as a surgeon, psychiatrist, or emergency medicine practitioner. We excluded non-specialist taxonomies, such as laboratories, ambulances, chiropractors, and physical and occupational therapists. The provider taxonomy and procedure codes we used to identify specialist visits are also listed in Table C.1 and Table C.2.

*Behavioral health visits.* We classified an in-person or telehealth claim as a behavioral health claim if (1) the service was delivered by a behavioral health provider, such as a psychiatrist, psychologist, therapist, social worker, or primary care provider, and (2) it included a behavioral health diagnosis or procedure code. The provider taxonomy and procedure codes we used to classify behavioral health claims are listed in Table C.1 and Table C.3.

**Table C.1.** Provider taxonomy codes used to identify primary care, specialty care, and behavioral health providers.

| Type | Code | Description | Primary Care | Specialty Care | Behavioral Health |
| --- | --- | --- | --- | --- | --- |
| PECOS | 14-50 | Nurse Practitioner | Yes | No | Yes |
| PECOS | 14-97 | Physician Assistant | Yes | No | Yes |
| PECOS | 14-01 | Practitioner - General Practice | Yes | No | Yes |
| PECOS | 14-02 | Practitioner - General Surgery | No | Yes | No |
| PECOS | 14-03 | Practitioner - Allergy/Immunology | No | Yes | No |
| PECOS | 14-04 | Practitioner - Otolaryngology | No | Yes | No |
| PECOS | 14-05 | Practitioner - Anesthesiology | No | Yes | No |
| PECOS | 14-06 | Practitioner - Cardiovascular Disease (Cardiology) | No | Yes | No |
| PECOS | 14-07 | Practitioner - Dermatology | No | Yes | No |
| PECOS | 14-08 | Practitioner - Family Practice | Yes | No | Yes |
| PECOS | 14-09 | Practitioner - Interventional Pain Management | No | Yes | No |
| PECOS | 14-10 | Practitioner - Gastroenterology | No | Yes | No |
| PECOS | 14-11 | Practitioner - Internal Medicine | Yes | No | Yes |
| PECOS | 14-12 | Practitioner - Osteopathic Manipulative Medicine | No | Yes | No |
| PECOS | 14-13 | Practitioner - Neurology | No | Yes | No |
| PECOS | 14-14 | Practitioner - Neurosurgery | No | Yes | No |
| PECOS | 14-16 | Practitioner - Obstetrics/Gynecology | Yes | No | No |
| PECOS | 14-17 | Practitioner - Hospice/Palliative Care | No | Yes | No |
| PECOS | 14-18 | Practitioner - Ophthalmology | No | Yes | No |
| PECOS | 14-19 | Practitioner - Oral Surgery | No | Yes | No |
| PECOS | 14-20 | Practitioner - Orthopedic Surgery | No | Yes | No |
| PECOS | 14-21 | Practitioner - Cardiac Electrophysiology | No | Yes | No |
| PECOS | 14-22 | Practitioner - Pathology | No | Yes | No |
| PECOS | 14-23 | Practitioner - Sports Medicine | No | Yes | No |
| PECOS | 14-24 | Practitioner - Plastic and Reconstructive Surgery | No | Yes | No |
| PECOS | 14-25 | Practitioner - Physical Medicine and Rehabilitation | No | Yes | No |
| PECOS | 14-26 | Practitioner - Psychiatry | No | Yes | Yes |
| PECOS | 14-27 | Practitioner - Geriatric Psychiatry | No | Yes | Yes |
| PECOS | 14-28 | Practitioner - Colorectal Surgery (Proctology) | No | Yes | No |
| PECOS | 14-29 | Practitioner - Pulmonary Disease | No | Yes | No |
| PECOS | 14-30 | Practitioner - Diagnostic Radiology | No | Yes | No |
| PECOS | 14-33 | Practitioner - Thoracic Surgery | No | Yes | No |
| PECOS | 14-34 | Practitioner - Urology | No | Yes | No |
| PECOS | 14-35 | Practitioner - Chiropractic | No | Yes | No |
| PECOS | 14-36 | Practitioner - Nuclear Medicine | No | Yes | No |
| PECOS | 14-37 | Practitioner - Pediatric Medicine | Yes | No | Yes |
| PECOS | 14-38 | Practitioner - Geriatric Medicine | Yes | No | Yes |
| PECOS | 14-39 | Practitioner - Nephrology | No | Yes | No |
| PECOS | 14-40 | Practitioner - Hand Surgery | No | Yes | No |
| PECOS | 14-41 | Practitioner - Optometry | No | Yes | No |
| PECOS | 14-44 | Practitioner - Infectious Disease | No | Yes | No |
| PECOS | 14-46 | Practitioner - Endocrinology | No | Yes | No |
| PECOS | 14-48 | Practitioner - Podiatry | No | Yes | No |
| PECOS | 14-66 | Practitioner - Rheumatology | No | Yes | No |
| PECOS | 14-72 | Practitioner - Pain Management | No | Yes | No |
| PECOS | 14-76 | Practitioner - Peripheral Vascular Disease | No | Yes | No |
| PECOS | 14-77 | Practitioner - Vascular Surgery | No | Yes | No |
| PECOS | 14-78 | Practitioner - Cardiac Surgery | No | Yes | No |
| PECOS | 14-79 | Practitioner - Addiction Medicine | No | Yes | Yes |
| PECOS | 14-81 | Practitioner - Critical Care (Intensivists) | No | Yes | No |
| PECOS | 14-82 | Practitioner - Hematology | No | Yes | No |
| PECOS | 14-83 | Practitioner - Hematology/Oncology | No | Yes | No |
| PECOS | 14-84 | Practitioner - Preventive Medicine | Yes | No | Yes |
| PECOS | 14-85 | Practitioner - Maxillofacial Surgery | No | Yes | No |
| PECOS | 14-86 | Practitioner - Neuropsychiatry | No | Yes | Yes |
| PECOS | 14-90 | Practitioner - Medical Oncology | No | Yes | No |
| PECOS | 14-91 | Practitioner - Surgical Oncology | No | Yes | No |
| PECOS | 14-92 | Practitioner - Radiation Oncology | No | Yes | No |
| PECOS | 14-93 | Practitioner - Emergency Medicine | No | Yes | No |
| PECOS | 14-94 | Practitioner - Interventional Radiology | No | Yes | No |
| PECOS | 14-98 | Practitioner - Gynecological Oncology | No | Yes | No |
| PECOS | 14-C0 | Practitioner - Sleep Medicine | No | Yes | No |
| PECOS | 14-C3 | Practitioner - Interventional Cardiology | No | Yes | No |
| PECOS | 14-C5 | Practitioner - Dentist | No | Yes | No |
| PECOS | 14-C6 | Practitioner - Hospitalist | No | Yes | No |
| PECOS | 14-C7 | Practitioner - Advanced Heart Failure and Transplant Cardiology | No | Yes | No |
| PECOS | 14-C8 | Practitioner - Medical Toxicology | No | Yes | No |
| PECOS | 14-C9 | Practitioner - Hematopoietic Cell Transplantation and Cellular Therapy | No | Yes | No |
| PECOS | 14-D3 | Practitioner - Medical Genetics and Genomics | No | Yes | No |
| PECOS | 14-D4 | Practitioner - Undersea and Hyperbaric Medicine | No | Yes | No |
| PECOS | 14-D7 | Practitioner - Micrographic Dermatologic Surgery (MDS) | No | Yes | No |
| PECOS | 14-D8 | Practitioner - Adult Congenital Heart Disease (ACHD) | No | Yes | No |
| PECOS | 14-62 | Psychologist, Clinical | No | No | Yes |
| PECOS | 14-68 | Psychologist, Clinical | No | No | Yes |
| PECOS | 14-80 | Licensed Clinical Social Worker | No | No | Yes |
| NPPES | 207Q00000X | Allopathic & Osteopathic Physicians - Family Medicine - Family Medicine | Yes | No | Yes |
| NPPES | 207QA0505X | Allopathic & Osteopathic Physicians - Family Medicine - Adult Medicine | Yes | No | Yes |
| NPPES | 207QG0300X | Allopathic & Osteopathic Physicians - Family Medicine - Geriatric Medicine | Yes | No | Yes |
| NPPES | 207QH0002X | Allopathic & Osteopathic Physicians - Family Medicine - Hospice and Palliative Medicine | Yes | No | Yes |
| NPPES | 208D00000X | Allopathic & Osteopathic Physicians - General Practice | Yes | No | Yes |
| NPPES | 207R00000X | Allopathic & Osteopathic Physicians - Internal Medicine | Yes | No | Yes |
| NPPES | 207RG0300X | Allopathic & Osteopathic Physicians - Internal Medicine - Geriatric Medicine | Yes | No | Yes |
| NPPES | 207RH0002X | Allopathic & Osteopathic Physicians - Internal Medicine - Hospice and Palliative Medicine | Yes | No | Yes |
| NPPES | 364S00000X | Physician Assistants & Advanced Practice Nursing Providers - Clinical Nurse Specialist | Yes | No | Yes |
| NPPES | 364SA2100X | Physician Assistants & Advanced Practice Nursing Providers - Clinical Nurse Specialist - Acute Care | Yes | No | Yes |
| NPPES | 364SA2200X | Physician Assistants & Advanced Practice Nursing Providers - Clinical Nurse Specialist - Adult Health | Yes | No | Yes |
| NPPES | 364SC2300X | Physician Assistants & Advanced Practice Nursing Providers - Clinical Nurse Specialist - Chronic Care | Yes | No | Yes |
| NPPES | 364SC1501X | Physician Assistants & Advanced Practice Nursing Providers - Clinical Nurse Specialist - Community Health/Public Health | Yes | No | Yes |
| NPPES | 364SF00001X | Physician Assistants & Advanced Practice Nursing Providers - Clinical Nurse Specialist - Family Health | Yes | No | Yes |
| NPPES | 364SG0600X | Physician Assistants & Advanced Practice Nursing Providers - Clinical Nurse Specialist - Gerontology | Yes | No | Yes |
| NPPES | 364SH1100X | Physician Assistants & Advanced Practice Nursing Providers - Clinical Nurse Specialist - Holistic | Yes | No | Yes |
| NPPES | 364SW0102X | Physician Assistants & Advanced Practice Nursing Providers - Clinical Nurse Specialist - Women’s Health | Yes | No | Yes |
| NPPES | 363L00000X | Physician Assistants & Advanced Practice Nursing Providers - Nurse Practitioner | Yes | No | Yes |
| NPPES | 363LA2200X | Physician Assistants & Advanced Practice Nursing Providers - Nurse Practitioner - Adult Health | Yes | No | Yes |
| NPPES | 363LC1500X | Physician Assistants & Advanced Practice Nursing Providers - Nurse Practitioner - Community Health | Yes | No | Yes |
| NPPES | 363LF0000X | Physician Assistants & Advanced Practice Nursing Providers - Nurse Practitioner - Family | Yes | No | Yes |
| NPPES | 363LG0600X | Physician Assistants & Advanced Practice Nursing Providers - Nurse Practitioner - Gerontology | Yes | No | Yes |
| NPPES | 363LP2300X | Physician Assistants & Advanced Practice Nursing Providers - Nurse Practitioner - Primary Care | Yes | No | Yes |
| NPPES | 363LW0102X | Physician Assistants & Advanced Practice Nursing Providers - Nurse Practitioner - Women’s Health | Yes | No | Yes |
| NPPES | 363A00000X | Physician Assistants & Advanced Practice Nursing Providers - Physician Assistant | Yes | No | Yes |
| NPPES | 363AM0700X | Physician Assistants & Advanced Practice Nursing Providers - Physician Assistant - Medical | Yes | No | Yes |
| NPPES | 363LP0808X | Physician Assistants & Advanced Practice Nursing Providers - Nurse Practitioner - Psychiatric/Mental Health | No | Yes | Yes |
| NPPES | 102L00000X | Behavioral Health & Social Service Providers - Psychoanalyst | No | No | Yes |
| NPPES | 103TA0400X | Behavioral Health & Social Service Providers - Psychologist - Addiction (substance use disorder) | No | No | Yes |
| NPPES | 103TA0700X | Behavioral Health & Social Service Providers - Psychologist - Adult Development & Aging | No | No | Yes |
| NPPES | 103TB0200X | Behavioral Health & Social Service Providers - Psychologist - Cognitive & Behavioral | No | No | Yes |
| NPPES | 103TC1900X | Behavioral Health & Social Service Providers - Psychologist - Counseling | No | No | Yes |
| NPPES | 103TE1000X | Behavioral Health & Social Service Providers - Psychologist - Educational | No | No | Yes |
| NPPES | 103TE1100X | Behavioral Health & Social Service Providers - Psychologist - Exercise & Sports | No | No | Yes |
| NPPES | 103TF0000X | Behavioral Health & Social Service Providers - Psychologist - Family | No | No | Yes |
| NPPES | 103TF0200X | Behavioral Health & Social Service Providers - Psychologist - Forensic | No | No | Yes |
| NPPES | 103TH0004X | Behavioral Health & Social Service Providers - Psychologist - Health | No | No | Yes |
| NPPES | 103TH0100X | Behavioral Health & Social Service Providers - Psychologist - Health Service | No | No | Yes |
| NPPES | 103TM1700X | Behavioral Health & Social Service Providers - Psychologist - Men & Masculinity | No | No | Yes |
| NPPES | 103TM1800X | Behavioral Health & Social Service Providers - Psychologist - Mental Retardation & Developmental Disabilities | No | No | Yes |
| NPPES | 103TP0016X | Behavioral Health & Social Service Providers - Psychologist - Prescribing (Medical) | No | No | Yes |
| NPPES | 103TP0814X | Behavioral Health & Social Service Providers - Psychologist - Psychoanalysis | No | No | Yes |
| NPPES | 103TP2700X | Behavioral Health & Social Service Providers - Psychologist - Psychotherapy | No | No | Yes |
| NPPES | 103TP2701X | Behavioral Health & Social Service Providers - Psychologist - Group Psychotherapy | No | No | Yes |
| NPPES | 103TR0400X | Behavioral Health & Social Service Providers - Psychologist - Rehabilitation | No | No | Yes |
| NPPES | 103TW0100X | Behavioral Health & Social Service Providers - Psychologist - Women^ | No | No | Yes |
| NPPES | 103TC0700X | Behavioral Health & Social Service Providers - Psychologist - Clinical | No | No | Yes |
| NPPES | 173F00000X | Behavioral Health & Social Service Providers - Psychologist - Sleep Specialist, PhD | No | No | Yes |
| NPPES | 103G00000X | Behavioral Health & Social Service Providers - Clinical Neuropsychologist | No | No | Yes |
| NPPES | 106H00000X | Behavioral Health & Social Service Providers - Marriage & Family Therapist | No | No | Yes |
| NPPES | 102X00000X | Behavioral Health & Social Service Providers - Poetry Therapist | No | No | Yes |
| NPPES | 222Q00000X | Respiratory, Developmental, Rehabilitative and Restorative Service Providers - Developmental Therapist | No | No | Yes |
| NPPES | 225A00000X | Respiratory, Developmental, Rehabilitative and Restorative Service Providers - Music Therapist | No | No | Yes |
| NPPES | 225800000X | Respiratory, Developmental, Rehabilitative and Restorative Service Providers - Recreation Therapist | No | No | Yes |
| NPPES | 225600000X | Respiratory, Developmental, Rehabilitative and Restorative Service Providers - Dance Therapist | No | No | Yes |
| NPPES | 221700000X | Respiratory, Developmental, Rehabilitative and Restorative Service Providers - Art Therapist | No | No | Yes |
| NPPES | 225700000X | Respiratory, Developmental, Rehabilitative and Restorative Service Providers - Massage Therapist | No | No | Yes |
| NPPES | 226000000X | Respiratory, Developmental, Rehabilitative and Restorative Service Providers - Recreation Therapist | No | No | Yes |
| NPPES | 101Y00000X | Behavioral Health & Social Service Providers - Counselor | No | No | Yes |
| NPPES | 101YM0800X | Behavioral Health & Social Service Providers - Counselor - Mental Health | No | No | Yes |
| NPPES | 101YA0400X | Behavioral Health & Social Service Providers - Counselor - substance use disorder/Addiction | No | No | Yes |
| NPPES | 225C00000X | Behavioral Health & Social Service Providers - Counselor - Rehabilitation Counselor | No | No | Yes |
| NPPES | 101YP1600X | Behavioral Health & Social Service Providers - Counselor - Pastoral | No | No | Yes |
| NPPES | 101YP2500X | Behavioral Health & Social Service Providers - Counselor - Professional | No | No | Yes |
| NPPES | 101YS0200X | Behavioral Health & Social Service Providers - Counselor - School | No | No | Yes |
| NPPES | 364SN0800X | Physician Assistants & Advanced Practice Nursing Providers - Clinical Nurse Specialist - Neuroscience | No | No | Yes |
| NPPES | 364SP0808X | Physician Assistants & Advanced Practice Nursing Providers - Clinical Nurse Specialist - Psychiatric/Mental Health | No | Yes | Yes |
| NPPES | 364SP0809X | Physician Assistants & Advanced Practice Nursing Providers - Nurse Practitioner - Psychiatric/Mental Health, Adult | No | Yes | Yes |
| NPPES | 364SP0811X | Physician Assistants & Advanced Practice Nursing Providers – Clinical Nurse Specialist - Psychiatric/Mental Health, Chronically Ill | No | Yes | Yes |
| NPPES | 364SP0812X | Physician Assistants & Advanced Practice Nursing Providers - Clinical Nurse Specialist - Psychiatric/Mental Health, Community | No | Yes | Yes |
| NPPES | 364SP0813X | Physician Assistants & Advanced Practice Nursing Providers - Clinical Nurse Specialist - Psychiatric/Mental Health, Geropsychiatric | No | Yes | Yes |
| NPPES | 163WP0808X | Physician Assistants & Advanced Practice Nursing Providers - Clinical Nurse Specialist - Psychiatric/Mental Health | No | Yes | Yes |
| NPPES | 163WP0809X | Physician Assistants & Advanced Practice Nursing Providers - Clinical Nurse Specialist -Psychiatric/Mental Health, Adult | No | Yes | Yes |
| NPPES | 163WA0400X | Nursing Service Providers – Registered Nurse - Addiction (substance use disorder) | No | Yes | Yes |
| NPPES | 163WP0000X | Nursing Service Providers – Registered Nurse - Pain Management | No | Yes | Yes |
| NPPES | 225XN1300X | Respiratory, Developmental, Rehabilitative and Restorative Service providers – Occupational Therapist - Neurorehabilitation | No | Yes | Yes |
| NPPES | 225XM0800X | Respiratory, Developmental, Rehabilitative and Restorative Service providers – Occupational Therapist - Mental Health Specialization | No | Yes | Yes |
| NPPES | 207RA0401X | Allopathic & Osteopathic Physicians - Internal Medicine - Addiction Medicine | No | Yes | Yes |
| NPPES | 207QS1201X | Allopathic & Osteopathic Physicians - Family Medicine - Sleep Medicine Specialization | No | Yes | No |
| NPPES | 207QA0401X | Allopathic & Osteopathic Physicians - Family Medicine - Addiction Medicine | No | Yes | Yes |
| NPPES | 2084N0600X | Allopathic & Osteopathic Physicians - Psychiatry & Neurology - Clinical Neurophysiology | No | Yes | No |
| NPPES | 2084N0400X | Allopathic & Osteopathic Physicians - Psychiatry & Neurology - Neurology | No | Yes | No |
| NPPES | 2084N0402X | Allopathic & Osteopathic Physicians - Psychiatry & Neurology - Neurology with Special Qualifications in Child Neurology | No | Yes | No |
| NPPES | 207T00000X | Allopathic & Osteopathic Physicians - Psychiatry & Neurology - Neurological Surgery | No | Yes | No |
| NPPES | 2084N0008X | Allopathic & Osteopathic Physicians - Psychiatry & Neurology - Neuromuscular Medicine | No | Yes | No |
| NPPES | 2084P0005X | Allopathic & Osteopathic Physicians - Psychiatry & Neurology - Neurodevelopmental Disabilities | No | Yes | Yes |
| NPPES | 2084P0015X | Allopathic & Osteopathic Physicians - Psychiatry & Neurology - Psychosomatic Medicine | No | Yes | Yes |
| NPPES | 2084P2900X | Allopathic & Osteopathic Physicians - Psychiatry & Neurology - Pain Medicine | No | Yes | Yes |
| NPPES | 2084S0010X | Allopathic & Osteopathic Physicians - Psychiatry & Neurology - Sports Medicine | No | Yes | Yes |
| NPPES | 2084S0012X | Allopathic & Osteopathic Physicians - Psychiatry & Neurology - Sleep Medicine | No | Yes | Yes |
| NPPES | 2084V0102X | Allopathic & Osteopathic Physicians - Psychiatry & Neurology - Vascular Neurology | No | Yes | No |
| NPPES | 2084A2900X | Allopathic & Osteopathic Physicians - Psychiatry & Neurology - Neurocritical Care | No | Yes | No |
| NPPES | 2084B0002X | Allopathic & Osteopathic Physicians - Psychiatry & Neurology - Bariatric Medicine | No | Yes | No |
| NPPES | 2084P0301X | Allopathic & Osteopathic Physicians - Psychiatry & Neurology - Brain Injury Medicine | No | Yes | Yes |
| NPPES | 2084F0202X | Allopathic & Osteopathic Physicians - Psychiatry & Neurology - Forensic Psychiatry | No | Yes | Yes |
| NPPES | 2084H0002X | Allopathic & Osteopathic Physicians - Psychiatry & Neurology - Hospice and Palliative Medicine | No | Yes | No |
| NPPES | 2084P0800X | Allopathic & Osteopathic Physicians - Psychiatry & Neurology - Psychiatry | No | Yes | Yes |
| NPPES | 2084P0802X | Allopathic & Osteopathic Physicians - Psychiatry & Neurology - Addiction Psychiatry | No | Yes | Yes |
| NPPES | 2084P0805X | Allopathic & Osteopathic Physicians - Psychiatry & Neurology - Geriatric Psychiatry | No | Yes | Yes |
| NPPES | 2084B0040X | Allopathic & Osteopathic Physicians - Psychiatry & Neurology - Behavioral Neurology & Neuropsychiatry Specialty | No | Yes | Yes |
| NPPES | 2084D0003X | Allopathic & Osteopathic Physicians - Psychiatry & Neurology - Diagnostic Neuroimaging | No | Yes | No |
| NPPES | 2084A0401X | Allopathic & Osteopathic Physicians - Psychiatry & Neurology - Addiction Medicine | No | Yes | Yes |
| NPPES | 2083A0300X | Allopathic & Osteopathic Physicians - Preventive Medicine - Addiction Medicine | No | Yes | Yes |
| NPPES | 1041C0700X | Behavioral Health & Social Service Providers - Social Worker - Clinical | No | No | Yes |
| NPPES | 1041S0200X | Behavioral Health & Social Service Providers - Social Worker - School | No | No | Yes |
| NPPES | 103T00000X | Behavioral Health & Social Service Providers - Social Worker - Psychologist | No | No | Yes |
| NPPES | 103TS0200X | Behavioral Health & Social Service Providers - Social Worker - School | No | No | Yes |
| NPPES | 208600000X | Allopathic & Osteopathic Physicians - Surgery (General) | No | Yes | No |
| NPPES | 2086S0122X | Allopathic & Osteopathic Physicians - Surgery - Plastic and Reconstructive Surgery | No | Yes | No |
| NPPES | 2086S0105X | Allopathic & Osteopathic Physicians - Surgery - Surgery of the Hand | No | Yes | No |
| NPPES | 2086S0102X | Allopathic & Osteopathic Physicians - Surgery - Surgical Critical Care | No | Yes | No |
| NPPES | 2086X0206X | Allopathic & Osteopathic Physicians - Surgery - Surgical Oncology | No | Yes | No |
| NPPES | 2086S0127X | Allopathic & Osteopathic Physicians - Surgery - Trauma Surgery | No | Yes | No |
| NPPES | 2086S0129X | Allopathic & Osteopathic Physicians - Surgery - Vascular Surgery | No | Yes | No |
| NPPES | 208G00000X | Allopathic & Osteopathic Physicians - Surgery - Thoracic Surgery (Cardiothoracic Vascular Surgery) | No | Yes | No |
| NPPES | 204F00000X | Allopathic & Osteopathic Physicians - Surgery - Transplant Surgery | No | Yes | No |
| NPPES | 208C00000X | Allopathic & Osteopathic Physicians - Surgery - Colon & Rectal Surgery | No | Yes | No |
| NPPES | 204E00000X | Allopathic & Osteopathic Physicians - Oral & Maxillofacial Surgery | No | Yes | No |
| NPPES | 2086H0002X | Allopathic & Osteopathic Physicians - Surgery - Hospice and Palliative Care | No | Yes | No |
| NPPES | 207X00000X | Allopathic & Osteopathic Physicians - Orthopedic Surgery | No | Yes | No |
| NPPES | 207XS0114X | Allopathic & Osteopathic Physicians - Orthopedic Surgery - Adult Reconstructive Orthopedic Surgery | No | Yes | No |
| NPPES | 207XX0004X | Allopathic & Osteopathic Physicians - Orthopedic Surgery - Foot and Ankle Surgery | No | Yes | No |
| NPPES | 207XS0106X | Allopathic & Osteopathic Physicians - Orthopedic Surgery - Hand Surgery | No | Yes | No |
| NPPES | 207XS0117X | Allopathic & Osteopathic Physicians - Orthopedic Surgery - Orthopedic Surgery of the Spine | No | Yes | No |
| NPPES | 207XX0801X | Allopathic & Osteopathic Physicians - Orthopedic Surgery - Orthopedic Trauma | No | Yes | No |
| NPPES | 207XX0005X | Allopathic & Osteopathic Physicians - Orthopedic Surgery - Sports Medicine | No | Yes | No |
| NPPES | 208200000X | Allopathic & Osteopathic Physicians - Plastic Surgery | No | Yes | No |
| NPPES | 2082S0099X | Allopathic & Osteopathic Physicians - Plastic Surgery - Plastic Surgery Within the Head & Neck | No | Yes | No |
| NPPES | 2082S0105X | Allopathic & Osteopathic Physicians - Plastic Surgery - Surgery of the Hand | No | Yes | No |
| NPPES | 207Y00000X | Allopathic & Osteopathic Physicians - Otolaryngology | No | Yes | No |
| NPPES | 207YS0123X | Allopathic & Osteopathic Physicians - Otolaryngology - Facial Plastic Surgery | No | Yes | No |
| NPPES | 207YX0602X | Allopathic & Osteopathic Physicians - Otolaryngology - Otolaryngic Allergy | No | Yes | No |
| NPPES | 207YX0901X | Allopathic & Osteopathic Physicians - Otolaryngology - Otology &Neurotology | No | Yes | No |
| NPPES | 207YX0007X | Allopathic & Osteopathic Physicians - Otolaryngology - Plastic Surgery within the Head & Neck | No | Yes | No |
| NPPES | 207YX0905X | Allopathic & Osteopathic Physicians - Otolaryngology - Facial Plastic Surgery | No | Yes | No |
| NPPES | 207L00000X | Allopathic & Osteopathic Physicians - Anesthesiology | No | Yes | No |
| NPPES | 207LC0200X | Allopathic & Osteopathic Physicians - Anesthesiology - Critical Care Medicine | No | Yes | No |
| NPPES | 207RC0000X | Allopathic & Osteopathic Physicians – Internal Medicine – Cardiovascular Disease | No | Yes | No |
| NPPES | 207RG0100X | Allopathic & Osteopathic Physicians – Internal Medicine – Gastroenterology | No | Yes | No |
| NPPES | 207RP1001X | Allopathic & Osteopathic Physicians – Internal Medicine – Pulmonary Disease | No | Yes | No |
| NPPES | 207RN0300X | Allopathic & Osteopathic Physicians – Internal Medicine – Nephrology | No | Yes | No |
| NPPES | 207RI0200X | Allopathic & Osteopathic Physicians – Internal Medicine – Infectious Disease | No | Yes | No |
| NPPES | 207RE0101X | Allopathic & Osteopathic Physicians – Internal Medicine – Endocrinology, Diabetes, and Metabolism | No | Yes | No |
| NPPES | 207RR0500X | Allopathic & Osteopathic Physicians – Internal Medicine – Rheumatology | No | Yes | No |
| NPPES | 207RC0200X | Allopathic & Osteopathic Physicians – Internal Medicine – Critical Care Medicine | No | Yes | No |
| NPPES | 207RH0000X | Allopathic & Osteopathic Physicians – Internal Medicine – Hematology | No | Yes | No |
| NPPES | 207RH0003X | Allopathic & Osteopathic Physicians – Internal Medicine – Hematology & Oncology | No | Yes | No |
| NPPES | 207RX0202X | Allopathic & Osteopathic Physicians – Internal Medicine – Medical Oncology | No | Yes | No |
| NPPES | 207RB0002X | Allopathic & Osteopathic Physicians – Internal Medicine – Obesity Medicine | No | Yes | No |
| NPPES | 207RC0001X | Allopathic & Osteopathic Physicians – Internal Medicine – Clinical Cardiac Electrophysiology | No | Yes | No |
| NPPES | 207RH0005X | Allopathic & Osteopathic Physicians – Internal Medicine – Hypertension Specialist | No | Yes | No |
| NPPES | 207RI0001X | Allopathic & Osteopathic Physicians – Internal Medicine – Clinical & Laboratory Immunology | No | Yes | No |
| NPPES | 207RI0008X | Allopathic & Osteopathic Physicians – Internal Medicine – Hepatology | No | Yes | No |
| NPPES | 207RM1200X | Allopathic & Osteopathic Physicians – Internal Medicine – Magnetic Resonance Imaging (MRI) | No | Yes | No |
| NPPES | 207RS0010X | Allopathic & Osteopathic Physicians – Internal Medicine – Sports Medicine | No | Yes | No |
| NPPES | 207RT0003X | Allopathic & Osteopathic Physicians – Internal Medicine – Transplant Hepatology | No | Yes | No |
| NPPES | 207RA0001X | Allopathic & Osteopathic Physicians – Internal Medicine – Advanced Heart Failure and Transplant Cardiology | No | Yes | No |
| NPPES | 207RI0001X | Allopathic & Osteopathic Physicians – Internal Medicine – Clinical & Laboratory Immunology | No | Yes | No |
| NPPES | 207RI0011X | Allopathic & Osteopathic Physicians – Internal Medicine – Interventional Cardiology | No | Yes | No |
| NPPES | 207RA0001X | Allopathic & Osteopathic Physicians – Internal Medicine – Advanced Heart Failure and Transplant Cardiology | No | Yes | No |
| NPPES | 207N00000X | Allopathic & Osteopathic Physicians – Dermatology | No | Yes | No |
| NPPES | 207NI0002X | Allopathic & Osteopathic Physicians – Clinical & Laboratory Dermatological Immunology | No | Yes | No |
| NPPES | 207ND0101X | Allopathic & Osteopathic Physicians – MOHS-Micrographic Surgery | No | Yes | No |
| NPPES | 207ND0900X | Allopathic & Osteopathic Physicians – Dermapathology | No | Yes | No |
| NPPES | 207NS0135X | Allopathic & Osteopathic Physicians – Procedural Dermatology | No | Yes | No |
| NPPES | 207V00000X | Allopathic & Osteopathic Physicians - Obstetrics & Gynecology | Yes | No | No |
| NPPES | 207VB0002X | Allopathic & Osteopathic Physicians – Obstetrics & Gynecology - Obesity Medicine | No | Yes | No |
| NPPES | 207VC0200X | Allopathic & Osteopathic Physicians – Obstetrics & Gynecology - Critical Care Medicine | No | Yes | No |
| NPPES | 207VF0040X | Allopathic & Osteopathic Physicians – Obstetrics & Gynecology - Female Pelvic Medicine and Reconstructive Surgery | No | Yes | No |
| NPPES | 207VX0201X | Allopathic & Osteopathic Physicians – Obstetrics & Gynecology - Gynecologic Oncology | No | Yes | No |
| NPPES | 207VG0400X | Allopathic & Osteopathic Physicians - Obstetrics & Gynecology - Gynecology | Yes | No | No |
| NPPES | 207VX0000X | Allopathic & Osteopathic Physicians - Obstetrics & Gynecology - Obstetrics | Yes | No | No |
| NPPES | 207VE0102X | Allopathic & Osteopathic Physicians – Obstetrics & Gynecology - Reproductive Endocrinology | No | Yes | No |
| NPPES | 207VH0002X | Allopathic & Osteopathic Physicians – Obstetrics & Gynecology - Hospice and Palliative Medicine** | No | Yes | No |
| NPPES | 207W00000X | Allopathic & Osteopathic Physicians – Ophthalmology | No | Yes | No |
| NPPES | 207WX0009X | Allopathic & Osteopathic Physicians – Ophthalmology - Glaucoma Specialist | No | Yes | No |
| NPPES | 207WX0107X | Allopathic & Osteopathic Physicians – Ophthalmology - Retina Specialist | No | Yes | No |
| NPPES | 207WX0108X | Allopathic & Osteopathic Physicians – Ophthalmology - Uveitis and Ocular Inflammatory Disease | No | Yes | No |
| NPPES | 207WX0120X | Allopathic & Osteopathic Physicians – Ophthalmology - Cornea and External Diseases Specialist | No | Yes | No |
| NPPES | 207WX0200X | Allopathic & Osteopathic Physicians – Ophthalmology - Ophthalmic Plastic and Reconstructive Surgery | No | Yes | No |
| NPPES | 1223S0112X | Dental Providers – Dentist – Oral and Maxillofacial Surgery | No | Yes | No |
| NPPES | 207ZP0101X | Allopathic & Osteopathic Physicians – Pathology - Anatomic Pathology | No | Yes | No |
| NPPES | 207ZP0102X | Allopathic & Osteopathic Physicians – Pathology - Anatomic Pathology & Clinical Pathology | No | Yes | No |
| NPPES | 207ZP0104X | Allopathic & Osteopathic Physicians – Pathology - Chemical Pathology | No | Yes | No |
| NPPES | 207ZC0006X | Allopathic & Osteopathic Physicians – Pathology - Clinical Pathology | No | Yes | No |
| NPPES | 207ZP0105X | Allopathic & Osteopathic Physicians – Pathology - Laboratory Medicine | No | Yes | No |
| NPPES | 207ZC0500X | Allopathic & Osteopathic Physicians – Pathology - Cytopathology | No | Yes | No |
| NPPES | 207ZD0900X | Allopathic & Osteopathic Physicians – Pathology - Dermapathology | No | Yes | No |
| NPPES | 207ZF0201X | Allopathic & Osteopathic Physicians – Pathology - Forensic Pathology | No | Yes | No |
| NPPES | 207ZH0000X | Allopathic & Osteopathic Physicians – Pathology - Hematology | No | Yes | No |
| NPPES | 207ZI0100X | Allopathic & Osteopathic Physicians – Pathology - Immunopathology | No | Yes | No |
| NPPES | 207ZM0300X | Allopathic & Osteopathic Physicians – Pathology - Medical Microbiology | No | Yes | No |
| NPPES | 207ZP0007X | Allopathic & Osteopathic Physicians – Pathology - Molecular Genetic Pathology | No | Yes | No |
| NPPES | 208100000X | Allopathic & Osteopathic Physicians – Physical Medicine & Rehabilitation | No | Yes | No |
| NPPES | 2081P0301X | Allopathic & Osteopathic Physicians – Physical Medicine & Rehabilitation - Brain Injury Medicine | No | Yes | No |
| NPPES | 2081S0010X | Allopathic & Osteopathic Physicians – Physical Medicine & Rehabilitation -Sports Medicine | No | Yes | No |
| NPPES | 2081H0002X | Allopathic & Osteopathic Physicians – Physical Medicine & Rehabilitation - Hospice and Palliative Medicine | No | Yes | No |
| NPPES | 2081N0008X | Allopathic & Osteopathic Physicians – Physical Medicine & Rehabilitation - Neuromuscular Medicine | No | Yes | No |
| NPPES | 2081P2900X | Allopathic & Osteopathic Physicians – Physical Medicine & Rehabilitation - Pain Medicine | No | Yes | No |
| NPPES | 2081P0004X | Allopathic & Osteopathic Physicians – Physical Medicine & Rehabilitation - Spinal Cord Injury Medicine | No | Yes | No |
| NPPES | 208VP0000X | Allopathic & Osteopathic Physicians – Physical Medicine & Rehabilitation - Interventional Pain Medicine | No | Yes | No |
| NPPES | 1223X0008X | Allopathic & Osteopathic Physicians – Radiology | No | Yes | No |
| NPPES | 2085R0202X | Allopathic & Osteopathic Physicians – Radiology - Diagnostic Radiology | No | Yes | No |
| NPPES | 2085R0001X | Allopathic & Osteopathic Physicians – Radiology - Radiation Oncology | No | Yes | No |
| NPPES | 204C00000X | Allopathic & Osteopathic Physicians – Radiology - Nuclear Medicine Practitioner | No | Yes | No |
| NPPES | 207U00000X | Allopathic & Osteopathic Physicians – Nuclear Medicine | No | Yes | No |
| NPPES | 207UN0901X | Allopathic & Osteopathic Physicians – Nuclear Medicine - Nuclear Cardiology | No | Yes | No |
| NPPES | 207UN0902X | Allopathic & Osteopathic Physicians – Nuclear Medicine - Nuclear Medicine Practitioner | No | Yes | No |
| NPPES | 207UN0903X | Allopathic & Osteopathic Physicians – Nuclear Medicine - Nuclear Medicine Practitioner | No | Yes | No |
| NPPES | 2085B0100X | Allopathic & Osteopathic Physicians – Radiology - Body Imaging | No | Yes | No |
| NPPES | 2085D0003X | Allopathic & Osteopathic Physicians – Radiology - Diagnostic Neuroimaging | No | Yes | No |
| NPPES | 2085N0700X | Allopathic & Osteopathic Physicians – Radiology - Neuroradiology | No | Yes | No |
| NPPES | 2085N0904X | Allopathic & Osteopathic Physicians – Radiology - Nuclear Radiology | No | Yes | No |
| NPPES | 2085R0204X | Allopathic & Osteopathic Physicians – Radiology - Vascular & Interventional | No | Yes | No |
| NPPES | 2085R0205X | Allopathic & Osteopathic Physicians – Radiology - Radiological Physics | No | Yes | No |
| NPPES | 2085U0001X | Allopathic & Osteopathic Physicians – Radiology - Diagnostic Ultrasound | No | Yes | No |
| NPPES | 2085R0203X | Allopathic & Osteopathic Physicians – Radiology - Therapeutic Radiology | No | Yes | No |
| NPPES | 2085H0002X | Allopathic & Osteopathic Physicians – Radiology - Hospice and Palliative Medicine | No | Yes | No |
| NPPES | 208800000X | Allopathic & Osteopathic Physicians – Urology | No | Yes | No |
| NPPES | 2088F0040X | Allopathic & Osteopathic Physicians – Urology - Female Pelvic Medicine & Reconstructive Surgery | No | Yes | No |
| NPPES | 152W00000X | Eye and Vision Services Providers - Optometrist | No | Yes | No |
| NPPES | 152WC0802X | Eye and Vision Services Providers - Optometrist - Corneal and Contact Management | No | Yes | No |
| NPPES | 152WL0500X | Eye and Vision Services Providers - Optometrist - Low Vision Rehabilitation | No | Yes | No |
| NPPES | 152WX0102X | Eye and Vision Services Providers - Optometrist - Occupational Vision | No | Yes | No |
| NPPES | 152WS0006X | Eye and Vision Services Providers - Optometrist - Sports Vision | No | Yes | No |
| NPPES | 152WV0400X | Eye and Vision Services Providers - Optometrist - Vision Therapy | No | Yes | No |
| NPPES | 213E00000X | Podiatric Medicine & Surgery Service Providers – Podiatrist – General Practice | No | Yes | No |
| NPPES | 213ES0103X | Podiatric Medicine & Surgery Service Providers – Podiatrist – Foot & Ankle Surgery | No | Yes | No |
| NPPES | 213ES0131X | Podiatric Medicine & Surgery Service Providers – Podiatrist – Foot Surgery | No | Yes | No |
| NPPES | 213EG0000X | Podiatric Medicine & Surgery Service Providers – Podiatrist – General Practice | Yes | No | No |
| NPPES | 213EP1101X | Podiatric Medicine & Surgery Service Providers – Podiatrist – Primary Podiatric Medicine | No | Yes | No |
| NPPES | 213EP0504X | Podiatric Medicine & Surgery Service Providers – Podiatrist – Public Medicine | No | Yes | No |
| NPPES | 213ER0200X | Podiatric Medicine & Surgery Service Providers – Podiatrist – Radiology | No | Yes | No |
| NPPES | 213ES0000X | Podiatric Medicine & Surgery Service Providers – Podiatrist – Sports Medicine | No | Yes | No |
| NPPES | 207P00000X | Allopathic & Osteopathic Physicians - Emergency Medicine | No | Yes | No |
| NPPES | 207PE0004X | Allopathic & Osteopathic Physicians - Emergency Medicine - Emergency Medical Services | No | Yes | No |
| NPPES | 207PH0002X | Allopathic & Osteopathic Physicians - Emergency Medicine - Hospice and Palliative Medicine | No | Yes | No |
| NPPES | 207PS0010X | Allopathic & Osteopathic Physicians - Emergency Medicine - Sports Medicine | No | Yes | No |
| NPPES | 207PE0005X | Allopathic & Osteopathic Physicians - Emergency Medicine - Undersea and Hyperbaric Medicine | No | Yes | No |
| NPPES | 207PT0002X | Allopathic & Osteopathic Physicians - Emergency Medicine - Medical Toxicology | No | Yes | No |
| NPPES | 207K00000X | Allopathic & Osteopathic Physicians - Allergy and Immunology | No | Yes | No |
| NPPES | 207KA0200X | Allopathic & Osteopathic Physicians - Allergy and Immunology - Allergy | No | Yes | No |
| NPPES | 207KI0005X | Allopathic & Osteopathic Physicians - Allergy and Immunology - Clinical and Laboratory Immunology | No | Yes | No |
| NPPES | 207RA0201X | Allopathic & Osteopathic Physicians - Internal Medicine - Allergy & Immunology | No | Yes | No |
| NPPES | 207LH0002X | Allopathic & Osteopathic Physicians – Anesthesiology – Hospice and Palliative Medicine | No | Yes | No |
| NPPES | 207YS0012X | Allopathic & Osteopathic Physicians – Otolaryngology – Sleep Medicine | No | Yes | No |
| NPPES | 207QA0000X | Allopathic & Osteopathic Physicians - Family Medicine - Adolescent Medicine | Yes | No | Yes |
| NPPES | 207QB0002X | Allopathic & Osteopathic Physicians - Family Medicine - Obesity Medicine (Family Medicine) Physician | Yes | No | Yes |
| NPPES | 207RA0000X | Allopathic & Osteopathic Physicians - Internal Medicine - Adolescent Medicine | Yes | No | Yes |
| NPPES | 208000000X | Allopathic & Osteopathic Physicians - Pediatrics | Yes | No | Yes |
| NPPES | 2080A0000X | Allopathic & Osteopathic Physicians - Pediatrics - Adolescent Medicine | Yes | No | Yes |
| NPPES | 2080C0008X | Allopathic & Osteopathic Physicians - Pediatrics - Child Abuse Pediatrics | Yes | No | Yes |
| NPPES | 2080P0006X | Allopathic & Osteopathic Physicians - Pediatrics - Developmental - Behavioral Pediatrics | Yes | No | Yes |
| NPPES | 2080P0008X | Allopathic & Osteopathic Physicians - Pediatrics -Neurodevelopmental Disabilities | Yes | No | Yes |
| NPPES | 2080B0002X | Allopathic & Osteopathic Physicians - Pediatrics - Obesity Medicine | Yes | No | Yes |
| NPPES | 2084P0804X | Allopathic & Osteopathic Physicians - Psychiatry & Neurology - Child & Adolescent Psychiatry | No | No | Yes |
| NPPES | 103GC0700X | Behavioral Health & Social Service Providers - Clinical Neuropsychologist - Clinical (Deactivated) | No | No | Yes |
| NPPES | 103TC2200X | Behavioral Health & Social Service Providers - Psychologist - Clinical Child & Adolescent | No | No | Yes |
| NPPES | 104100000X | Behavioral Health & Social Service Providers - Social Worker | No | No | Yes |

Note: Provider types are not mutually exclusive. For example, certain primary care providers are also classified as behavioral health providers, and some specialty care providers are also classified as behavioral health providers. However, no primary care providers are classified as specialty care providers.

NPPES = National Plan and Provider Enumeration System; PECOS = (Medicare) Provider Enrollment, Chain, and Ownership System.

**Table C.2.** Procedure codes used to identify primary and specialty care visits.

| Code Type | Code | Description | POS restriction |
| --- | --- | --- | --- |
| Procedure (CPT) | 96160 | Administration of patient-focused health risk assessment instrument (e.g., health hazard appraisal) with scoring and documentation, per standardized instrument | No |
| Procedure (CPT) | 96161 | Administration of caregiver-focused health risk assessment instrument (e.g., depression inventory) for the benefit of the patient, with scoring and documentation, per standardized instrument | No |
| Procedure (CPT) | 98966-98968 | Telephone assessment and management service provided by a qualified non-physician health care professional to an established client, parent, or guardian | No |
| Procedure (CPT) | 98969-98972 | Online Digital Assessment and Management Service by Qualified Nonphysician Health Care Professional | No |
| Procedure (CPT) | 98980 | Remote Therapeutic Monitoring Treatment Management Services | No |
| Procedure (CPT) | 99091 | Remote Physiologic Patient Monitoring | No |
| Procedure (CPT) | 99421-99423 | Online digital E&M services - physicians or other qualified health professionals | No |
| Procedure (CPT) | 99441-99443 | Telephone E&M | No |
| Procedure (CPT) | 99444 | Online E&M (deleted in 2020 and replaced w/99421-99423) | No |
| Procedure (CPT) | 99453-99454 | Chronic Care Remote Patient Monitoring Codes | No |
| Procedure (CPT) | 99457 | Remote physiologic monitoring treatment management services | No |
| Procedure (CPT) | 99474 | Home blood pressure monitoring support (new in 2020) | No |
| Procedure (CPT) | 98975-98977 | Remote therapeutic monitoring services. Code 98975 represents the initial setup and patient education for the equipment. Codes 98976 (respiratory system) and 98977 (musculoskeletal system) represent the device supply w/ scheduled recording and/or programmed alert transmission for a 30-day period (new in 2022) | No |
| Procedure (CPT) | 99201 | Office or Other OP visit (99201 deleted in 2021) | No |
| Procedure (CPT) | 99202-99205 | Office or Other Outpatient Services – New Patient | No |
| Procedure (CPT) | 99211-99215 | Office or Other Outpatient Services – Established Patient | No |
| Procedure (CPT) | 99324-99328 | New Patient Visit in Domiciliary, Rest or Custodial Care | No |
| Procedure (CPT) | 99334-99337 | Established Patient Visit in Domiciliary, Rest or Custodial Care | No |
| Procedure (CPT) | 99339-99340 | Care plan oversight for a patient in home or domiciliary care | No |
| Procedure (CPT) | 99341-99345 | Home or residence E&M services, new patient | No |
| Procedure (CPT) | 99347-99350 | Home or residence E&M services, established patient | No |
| Procedure (CPT) | 99424, 99426 | Principal Care Management | No |
| Procedure (CPT) | 99429 | Other Preventive Medicine Services | No |
| Procedure (CPT) | 99439 | Chronic Care Management Services | No |
| Procedure (CPT) | 99483 | Cognitive Assessment and Care Plan Services | No |
| Procedure (CPT) | 99484 | General Behavioral Health Integration Care Management | No |
| Procedure (CPT) | 99487 | Complex Chronic Care Management Services | No |
| Procedure (CPT) | 99489 | Complex Chronic Care Management Services | No |
| Procedure (CPT) | 99490-99491 | Chronic Care Management Services | No |
| Procedure (CPT) | 99492 | Initial psychiatric collaborative care management, first 70 minutes in the first calendar month of  behavioral health care manager activities | No |
| Procedure (CPT) | 99493 | Subsequent psychiatric collaborative care management, first 60 minutes in a subsequent month of behavioral health care manager activities | No |
| Procedure (CPT) | 99494 | Initial or subsequent psychiatric collaborative care management, each additional 30 minutes in a calendar month of behavioral health care manager activities | No |
| Procedure (CPT) | 99495-99496 | Transitional care management (99495 - moderate medical complexity requiring a face-to-face visit within 14 days of discharge; 99496 - high medical complexity requiring a face-to-face visit within seven days of discharge) | Yes |
| Procedure (CPT) | 99497 | Advanced directive counseling and discussion | Yes |
| Procedure (CPT) | 99498 | Advance Care Planning | Yes |
| Procedure (CPT) | 99354-99355 | Prolonged Service with Direct Patient Contact (Except with Office or Other Outpatient Services) | No |
| Procedure (CPT) | 99358-99359 | Prolonged Service on Date Other Than the Face-to-Face Evaluation and Management Service Without Direct Patient Contact | Yes |
| Procedure (CPT) | 99415-99416 | Prolonged Clinical Staff Services with Physician or Other Qualified Health Care Professional Supervision | No |
| Procedure (HCPCS) | G0076-G0087 | Home Care Management Services | No |
| Procedure (HCPCS) | G0101 | Cervical or vaginal cancer screening; pelvic and clinical breast examination | No |
| Procedure (HCPCS) | G0102 | Prostate cancer screening; digital rectal examination (DRE) | No |
| Procedure (HCPCS) | G0108 | Diabetes outpatient self-management training services, individual, per 30 minutes | No |
| Procedure (HCPCS) | G0109 | Diabetes outpatient self-management training services, group session (2 or more), per 30 minutes | No |
| Procedure (HCPCS) | G0296 | Visit to determine lung cancer screening eligibility | No |
| Procedure (HCPCS) | G0402 | Welcome to Medicare visit | No |
| Procedure (HCPCS) | G0438-G0439 | Annual wellness visit; includes a personalized prevention plan of service (PPPS); initial and subsequent visits | No |
| Procedure (HCPCS) | G0442 | Annual alcohol misuse screening, 15 minutes | No |
| Procedure (HCPCS) | G0444 | Annual depression screening | No |
| Procedure (HCPCS) | G0502-G0504 | Psychiatric collaborative care management Other (Deleted in 2018 and replaced with 99492-99494) | No |
| Procedure (HCPCS) | G0506 | CCM service: Comprehensive assessment and care planning for patients needing chronic care | No |
| Procedure (HCPCS) | G0507 | CCM service: Care management services for behavioral health conditions (Deleted in 2018 and replaced with 99484) | No |
| Procedure (HCPCS) | G2010 | Remote evaluation of recorded video and/or images submitted by an established patient | No |
| Procedure (HCPCS) | G2012 | Virtual check-in by a physician or other qualified health care professional who can report E&M services | No |
| Procedure (HCPCS) | G2061-G2063 | Qualified nonphysician healthcare professional online assessment and management service, for an established patient | No |
| Procedure (HCPCS) | G2064 | Principal care management service at least 30 minutes – physician or other qualified health care professional | No |
| Procedure (HCPCS) | G2065 | Principal care management service at least 30 minutes – clinical staff time directed by a physician or other qualified health care professional | No |
| Procedure (HCPCS) | G2214 | Psychiatric Collaborative Care Management (new in 2021) | No |
| Procedure (HCPCS) | G2250 | Remote assessment of recorded video and/ or images submitted by an established patient (new in 2021) | No |
| Procedure (HCPCS) | G2251 | Brief communication technology-based service, e.g., virtual check-in, by a qualified health care professional who cannot report E&M services (new in 2021) | No |
| Procedure (HCPCS) | G2252 | Brief communication technology-based service, e.g., virtual check-in, by a physician or other qualified health care professional who can report E&M services (new in 2021) | No |
| Procedure (HCPCS) | G9978-G9986 | Remote in-home visit for the E&M of a patient (BPCI) (new in 2019) | No |
| Procedure (HCPCS) | G9987 | Bundled payments (BPCI advanced) model home visit for patient assessment (new in 2019) | No |
| Procedure (HCPCS) | Q0091 | Screening Papanicolaou smear; obtaining, preparing and conveyance of cervical or vaginal smear to lab | No |
| Procedure (HCPCS) | G2212 | Prolonged office/outpatient E&M services | No |
| Procedure (HCPCS) | 92002 | Ophthalmological services: medical examination and evaluation | No |
| Procedure (HCPCS) | 92004 | Ophthalmological services: medical examination and evaluation | No |
| Procedure (HCPCS) | 92012 | Ophthalmological services: medical examination and evaluation | No |
| Procedure (HCPCS) | 92014 | Ophthalmological services: medical examination and evaluation | No |
| Procedure (HCPCS) | G0071 | Payment for communication technology-based services for 5 minutes or more of a virtual (non-face-to-face) communication between a rural health clinic (RHC) or federally qualified health center (FQHC) practitioner and RHC or FQHC patient, or 5 minutes or more of remote evaluation of recorded video and/or images by an RHC or FQHC practitioner, occurring in lieu of an office visit | No |
| Procedure (HCPCS) | G0468 | FQHC visit, IPPE or AWV; a FQHC visit that includes an initial preventive physical examination (IPPE) or annual wellness visit (AWV) and includes a typical bundle of Medicare-covered services that would be furnished per diem to a patient receiving an IPPE or AWV as maintained by CMS | No |

POS = Place of service; CPT = Current Procedural Terminology; E&M = Evaluation and management; HCPCS = Healthcare Common Procedure Coding System; OP = Outpatient; CCM = Chronic Care Management; PPPS = Personalized Prevention Plan of Service; FQHC = Federally-Qualified Health Center; RHC = Rural Health Center; IPPE = Initial Preventive Physical Examination; AWV = Annual Wellness Visit; CMS = Centers for Medicare and Medicaid Services; NPPES = National Plan and Provider Enumeration System; PECOS = (Medicare) Provider Enrollment, Chain, and Ownership System.

**Table C.3.** Procedure and diagnosis codes used to identify behavioral health visits.

| Type | Code | Description | POS Restriction |
| --- | --- | --- | --- |
| Procedure (CPT) | 90791 | Psychiatric Diagnostic Interview Examination | Yes |
| Procedure (CPT) | 90792 | Psychiatric Diagnostic Interview Examination | Yes |
| Procedure (CPT) | 90832 | Psychotherapy | Yes |
| Procedure (CPT) | 90833 | Psychotherapy with evaluation and management (E&M) | Yes |
| Procedure (CPT) | 90834 | Psychotherapy | Yes |
| Procedure (CPT) | 90836 | Psychotherapy with evaluation and management (E&M) | Yes |
| Procedure (CPT) | 90837 | Psychotherapy | Yes |
| Procedure (CPT) | 90838 | Psychotherapy with evaluation and management (E&M) | Yes |
| Procedure (CPT) | 90839 | Psychotherapy for crisis | Yes |
| Procedure (CPT) | 90845 | Other Psychotherapy Procedures | Yes |
| Procedure (CPT) | 90846 | Family psychotherapy | Yes |
| Procedure (CPT) | 90847 | Family psychotherapy | Yes |
| Procedure (CPT) | 90849 | Family psychotherapy | Yes |
| Procedure (CPT) | 90853 | Group psychotherapy | Yes |
| Procedure (CPT) | 90865 | Narcosynthesis for psychiatric diagnostic and/or therapeutic purposes | Yes |
| Procedure (CPT) | 90870 | Electroconvulsive therapy | Yes |
| Procedure (CPT) | 90880 | Medical hypnotherapy | Yes |
| Procedure (CPT) | 90899 | Unlisted psychiatric service or procedure | Yes |
| Procedure (CPT) | 96105 | Assessment of Aphasia and Cognitive Performance Testing | Yes |
| Procedure (CPT) | 96116 | Neurobehavioral status exam | Yes |
| Procedure (CPT) | 96125 | Standardized cognitive performance testing | Yes |
| Procedure (CPT) | 96127 | Brief emotional/behavioral assessment (e.g., depression inventory, attention-deficit/hyperactivity disorder scale) | Yes |
| Procedure (CPT) | 96130 | Psychological testing evaluation services by physician or other qualified health care professional | Yes |
| Procedure (CPT) | 96132 | Neuropsychological testing evaluation services by physician or other qualified health care professional | Yes |
| Procedure (CPT) | 96136 | Psychological or neuropsychological test administration and scoring by physician or other qualified health care professional, two or more tests, any method | Yes |
| Procedure (CPT) | 96138 | Psychological or neuropsychological test administration and scoring by technician, two or more tests, any method | Yes |
| Procedure (CPT) | 96146 | Psychological or neuropsychological test administration, with single automated instrument via electronic platform | Yes |
| Procedure (CPT) | 96150 | Health and behavior assessment (e.g., health-focused clinical interview, behavioral observations, psychophysiological monitoring, health-oriented questionnaires) | Yes |
| Procedure (CPT) | 96151 | Health and behavior assessment (e.g., health-focused clinical interview, behavioral observations, psychophysiological monitoring, health-oriented questionnaires) | Yes |
| Procedure (CPT) | 96152 | Health and behavior intervention, individual | Yes |
| Procedure (CPT) | 96153 | Health and behavior intervention, group | Yes |
| Procedure (CPT) | 96154 | Health and behavior intervention, family | Yes |
| Procedure (CPT) | 96155 | Health and behavior intervention, family | Yes |
| Procedure (CPT) | 96156 | Health behavior assessment or re-assessment – new in 2020 | Yes |
| Procedure (CPT) | 96158 | Health and behavior intervention, individual - new in 2020 | Yes |
| Procedure (CPT) | 96164 | Health and behavior intervention, group - new in 2020 | Yes |
| Procedure (CPT) | 96167 | Health and behavior intervention, family - new in 2020 | Yes |
| Procedure (CPT) | 96170 | Health and behavior intervention, family - new in 2020 | Yes |
| Procedure (CPT) | 97129 | Therapeutic interventions that focus on cognitive function - initial 15 min - new in 2020 | Yes |
| Procedure (CPT) | 97151 | Behavior Identification Assessment, administered by QHP | Yes |
| Procedure (CPT) | 97152 | Behavior Identification Supporting Assessment, administered by one technician under the direction of QHP, | Yes |
| Procedure (CPT) | 97153 | Adaptive Behavior Treatment by Protocol, administered by technician under the direction of a QHP | Yes |
| Procedure (CPT) | 97154 | Group Adaptive Behavior Treatment by Protocol, administered by technician under direction of QHP | Yes |
| Procedure (CPT) | 97155 | Adaptive Behavior Treatment with Protocol Modification, administered by QHP | Yes |
| Procedure (CPT) | 97156 | Family Adaptive Behavior Treatment Guidance, administered by QHP | Yes |
| Procedure (CPT) | 97157 | Multiple-Family Group Adaptive Behavior Treatment Guidance, administered by QHP (without the patient present) | Yes |
| Procedure (CPT) | 97158 | Group Adaptive Behavior Treatment with Protocol Modification, administered by QHP face-to-face with multiple patients, | Yes |
| Procedure (CPT) | 99406 | Smoking and tobacco use cessation counseling visit, greater than 3 minutes up to 10 minutes | No |
| Procedure (CPT) | 99407 | Smoking and tobacco use cessation counseling visit, intensive, greater than 10 minutes | No |
| Procedure (CPT) | 99408 | Alcohol and/or substance (other than tobacco) abuse structured screening (e.g., AUDIT, DAST), and brief intervention (SBI) services | No |
| Procedure (CPT) | 99409 | Alcohol and/or substance (other than tobacco) abuse structured screening (e.g., AUDIT, DAST), and brief intervention (SBI) services | No |
| Procedure (CPT) | 99484 | Care management services for behavioral health conditions | No |
| Procedure (CPT) | 99492 | First 70 minutes in the first calendar month for behavioral health care manager activities | No |
| Procedure (CPT) | 99493 | First 60 minutes in a subsequent month for behavioral health care manager activities | No |
| Procedure (CPT) | 99494 | Each additional 30 minutes in a calendar month of behavioral health care manager activities listed | No |
| Procedure (CPT) | 0360T | Observational behavioral follow-up assessment | No |
| Procedure (CPT) | 0364T | Adaptive behavior treatment by protocol, administered by technician, face-to-face with one patient | No |
| Procedure (CPT) | 0365T | Adaptive behavior treatment by protocol, administered by technician, face-to-face with one patient, each additional 30 min | No |
| Procedure (CPT) | 0702T | Remote therapeutic monitoring of a standardized online digital cognitive behavioral therapy program ; supply and technical support – new in 2022 | No |
| Procedure (CPT) | 0703T | Remote therapeutic monitoring of a standardized online digital cognitive behavioral therapy program; physician or other qualified health care professional – new in 2022 | Yes |
| Procedure (CPT) | 90785 | Interactive complexity add-on | Yes |
| Procedure (CPT) | 90840 | Psychotherapy for crisis - additional 30 minutes | Yes |
| Procedure (CPT) | 96121 | Neurobehavioral status exam, each additional hour | Yes |
| Procedure (CPT) | 96131 | Psychological testing evaluation services by physician or other qualified health care professional, each additional hour | Yes |
| Procedure (CPT) | 96133 | Neuropsychological testing evaluation services by physician or other qualified health care professional, each additional hour | Yes |
| Procedure (CPT) | 96137 | Psychological or neuropsychological test administration and scoring by physician or other qualified health care professional, two or more tests, any method, additional hour | Yes |
| Procedure (CPT) | 96139 | Psychological or neuropsychological test administration and scoring by technician, two or more tests, any method, each additional hour | Yes |
| Procedure (CPT) | 96159 | Health and behavior intervention, individual additional 15 min- new in 2020 | Yes |
| Procedure (CPT) | 96165 | Health and behavior intervention, group additional 15 min- new in 2020 | Yes |
| Procedure (CPT) | 96168 | Health and behavior intervention, family additional 15 min- new in 2020 | Yes |
| Procedure (CPT) | 96171 | Health and behavior intervention, family additional 15 min- new in 2020 | Yes |
| Procedure (CPT) | 97130 | Therapeutic interventions that focus on cognitive function - each additional 15 min - new in 2020 | Yes |
| Procedure (HCPCS) | G0396 | Alcohol and/or substance (other than tobacco) abuse structured screening (e.g., AUDIT, DAST), and brief intervention (SBI) services | No |
| Procedure (HCPCS) | G0397 | Alcohol and/or substance (other than tobacco) abuse structured screening (e.g., AUDIT, DAST), and brief intervention (SBI) services | No |
| Procedure (HCPCS) | G0409 | Social work and psychological services, directly relating to and/or furthering the patient's rehabilitation goals | No |
| Procedure (HCPCS) | G0443 | Brief face-to-face behavioral counseling for alcohol misuse | No |
| Procedure (HCPCS) | G0445 | High intensity behavioral counseling to prevent sexually transmitted infection | No |
| Procedure (HCPCS) | G0446 | Annual, face-to-face intensive behavioral therapy for cardiovascular disease, individual, 15 minutes | No |
| Procedure (HCPCS) | G0447 | Face-to-face behavioral counseling for obesity, 15 minutes | No |
| Procedure (HCPCS) | G0473 | Face-to-face behavioral counseling for obesity, group (2–10), 30 minutes | No |
| Procedure (HCPCS) | G0502 | Psychiatric collaborative care management | No |
| Procedure (HCPCS) | G0503 | Psychiatric collaborative care management | No |
| Procedure (HCPCS) | G0504 | Psychiatric collaborative care management | No |
| Procedure (HCPCS) | G2011 | Alcohol and/or substance abuse structured assessment and brief intervention - new in 2019 | No |
| Procedure (HCPCS) | G2076 | Intake activities, including a physician assessment, - opioid treatment program - new in 2020 | Yes |
| Procedure (HCPCS) | G2086-G2088 | Office-based treatment for opioid use disorder - new in 2020 | No |
| Procedure (HCPCS) | G2214 | Psychiatric Collaborative Care Management (new in 2021) | No |
| Diagnosis (ICD-10) | F01-F09 | Mental disorders due to known physiological conditions | No |
| Diagnosis (ICD-10) | F10-F19 | Mental and behavioral disorders due to psychoactive substance use (substance use disorders) | No |
| Diagnosis (ICD-10) | F20-F29 | Schizophrenia, schizotypal, delusional, and other non-mood psychotic disorders | No |
| Diagnosis (ICD-10) | F30-F39 | Mood [affective] disorders | No |
| Diagnosis (ICD-10) | F40-F48 | Anxiety, dissociative, stress-related, somatoform and other nonpsychotic mental disorders | No |
| Diagnosis (ICD-10) | F50-F59 | Behavioral syndromes associated with physiological disturbances and physical factors | No |
| Diagnosis (ICD-10) | F60-F69 | Disorders of adult personality and behavior | No |
| Diagnosis (ICD-10) | F70-F79 | Intellectual disabilities | No |
| Diagnosis (ICD-10) | F80-F89 | Pervasive and specific developmental disorders | No |
| Diagnosis (ICD-10) | F90-F98 | Behavioral and emotional disorders with onset usually occurring in childhood and adolescence | No |
| Diagnosis (ICD-10) | F99 | Unspecified mental disorder | No |

POS = Place of service; CPT = Current Procedural Terminology; E&M = Evaluation and management; HCPCS = Healthcare Common Procedure Coding System; ICD = International Classification of Diseases, Functioning, and Disability; AUDIT = Alcohol Use Disorders Identification Test; DAST = Drug Abuse Screening Test.

Appendix D

Regression specification and explanatory variables

We estimated logistic regressions predicting telehealth use in 2022 as a function of patient and area characteristics in 2021. For each insurance type (commercial and Medicare Advantage), we estimated a separate logistic regression for each of the two binary outcomes of any telehealth use and any audio-only telehealth use where the patient and area characteristics defined in 2021 are the independent variables. This resulted in a total of four regressions (two types of insurance * two outcomes). To account for similarities in patient outcomes and characteristics within a ZIP Code, we clustered standard errors at the ZIP Code level. We report regression coefficients as odds ratios.

We estimated the following equation representing a logistic regression model, where the log-odds corresponding to the probability of a binary outcome being equal to one is modeled as a linear combination of patient and area-level characteristics:

$$log\left( \frac{p_{ic}}{1-p_{ic}} \right)=\beta_{0}+\beta_{1}.X_{i}+\beta_{2}.Z_{c}+\varepsilon_{ic}$$

In the equation above, *I* represents a patient and *c* represents an area or ZIP Code; $p_{ic}$ is the probability that a binary outcome ($Y_{ic})$ is equal to one for person *I* in Zip Code *c*; $\beta_{0}$is the intercept; $\beta_{1}$ and $\beta_{2}$ represent coefficients on sets of patient ($X_{i}$) and area-level control variables ($Z_{c}$), respectively; and $\varepsilon_{ic}$ is the random error term.

Table D.1 lists the explanatory variables used in the logistic regressions.

**Table D.1. Explanatory variables used in logistic regressions**

| Type of explanatory variable | Explanatory variables | Data source |
| --- | --- | --- |
| Patient demographics | - Age categories (<18, 18–44, and 45–64 [reference category] for commercially insured patients; 65–74 [reference category], 75–84, and 85+ for Medicare Advantage patients)^1^ - Indicator for male gender | - MN APCD |
| Patient chronic conditions and  patient risk scores | - Indicators for specific chronic and behavioral health conditions that were prevalent among at least 10 percent of patients in the commercial or MA populations (depression, diabetes, hypertension, lipid metabolism disorder, glaucoma, hypothyroidism, ischemic heart disease, low back pain, and persistent asthma) - Indicator for whether patient is at high risk of adverse outcomes (ACG risk score above 75th percentile of the distribution) | - Condition markers based on patients’ diagnoses and prescription fills in 2021 created by the ACG system in the MN APCD - ACG risk score available in the MN APCD; specifically, the standardized, prospective risk score capturing the predicted risk for total cost in 2021. |
| Area characteristics | - Indicator for an area being in the lowest decile of broadband access, based on the percentage of households with broadband access in a ZIP Code - Indicator for an area being in the highest quartile of poverty, based on the percentage of households in poverty in a ZIP Code - Indicator for an area being in the highest quartile of the percentage of BIPOC residents, based on the percentage of BIPOC residents in each ZIP Code - Indicator for metropolitan versus nonmetropolitan location based on RUCA codes created by the USDA.^2^ | - ZIP Code-level data on variables from the ACS (5-year estimates for 2021) - ZIP Code-level data on RUCA designation available in the MN APCD |
| Variation in health system capacity due to changes in COVID-19 incidence | Quarterly rate of COVID-19 hospitalizations in each county in 2021 | MDH |

^1^ The commercial and MA samples were restricted to patients under 65 years and those 65 or older, respectively.

^2^ RUCA codes are created by the U.S. Department of Agriculture’s Economic Research Service and are available at <https://www.ers.usda.gov/data-products/rural-urban-commuting-area-codes/>. We classified patients as residents of a metropolitan area if their ZIP Code corresponded to any of the following RUCA codes:

RUCA = 1: Metropolitan area core: primary flow within an urbanized area (UA)

RUCA = 2: Metropolitan area high commuting: primary flow 30 percent or more to a UA

RUCA = 3: Metropolitan area low commuting: primary flow 10 to 30 percent to a UA.

Patients in all other RUCA codes, including micropolitan areas, rural areas, or small towns, were classified as living in nonmetropolitan areas.

ACG = Adjusted Clinical Group^®^; ACS = American Community Survey; BIPOC = Black, indigenous, and people of color; MA = Medicare Advantage; MDH = Minnesota Department of Health; MN APCD = Minnesota All Payer Claims Database; RUCA = rural-urban commuting area; USDA = U.S. Department of Agriculture.

Table D.2 shows the distribution of the commercial and MA samples across the age categories before excluding the 65+ and under-65 patients from the commercial and MA samples, respectively.

**Table D.2. Distribution of commercially insured and MA patients across age categories**

| Commercially insured patients | | | Medicare Advantage patients | | |
| --- | --- | --- | --- | --- | --- |
| Age category | Number | Percent | Age category | Number | Percent |
| Patient is under 18 | 194,974 | 22.30% | Patient is under 65 | 14,273 | 4.13% |
| Patient is 18–44 | 327,903 | 37.51% | Patient is 65–74 | 184,575 | 53.34% |
| Patient is 45–64 | 325,638 | 37.25% | Patient is 75–84 | 112,631 | 32.55% |
| Patient is 65+ | 25,736 | 2.94% | Patient is 85+ | 34,531 | 9.98% |
| **Total** | **874,251** | **100.00%** | **Total** | **346,010** | **100.00%** |

Appendix E

Identifying the Analysis Sample

Tables E.1 and E.2 show the stepwise process used to define the analysis sample for the study – to identify the commercially insured and Medicare Advantage patient samples meeting all inclusion criteria – for examining telehealth use in 2022 as a function of patient and area characteristics in 2021.

**Table E.1.** Stepwise process for identifying analytic sample: commercially insured patients

| Step | Criteria for retaining patients in the commercial sample | Patients retained | % patients excluded |
| --- | --- | --- | --- |
| 1 | Commercial insurance | 1,652,711 | n.a. |
| 2 | Valid Minnesota ZIP Code | 1,634,128 | 1% |
| 3 | Enrolled for at least three months in both 2021 and 2022 | 1,221,070 | 25% |
| 4a | At least one commercial claim in 2022 | 1,034,888 | 15% |
| 4b | At least one commercial claim in both 2021 and 2022 | 958,459 | 7% |
| 5 | No missing data for patient or area-level characteristics | 874,251 | 9% |

Source: Mathematica analysis of data from MN APCD, Extract 26.

n.a. = not applicable.

**Table E.2.** Stepwise process for identifying analytic sample: Medicare Advantage patients

| Step | Criteria for retaining patients in the Medicare Advantage sample | Patients retained | % patients excluded |
| --- | --- | --- | --- |
| 1 | Medicare Advantage insurance | 493,411 | n.a. |
| 2 | Valid Minnesota ZIP Code | 491,950 | 0.3% |
| 3 | Enrolled for at least three months in both 2021 and 2022 | 412,524 | 16% |
| 4a | At least one Medicare Advantage claim in 2022 | 384,014 | 7% |
| 4b | At least one Medicare Advantage claim in both 2021 and 2022 | 353,420 | 8% |
| 5 | No missing data for patient or area-level characteristics | 346,010 | 2% |

Source: Mathematica analysis of data from MN APCD, Extract 26.

n.a. = not applicable.

1. Minnesota Department of Health. “Minnesota All Payer Claims Database Extract 25 Overview.” <https://www.health.state.mn.us/data/apcd/docs/mnapcdoverview.pdf>. Accessed August 25, 2023. [↑](#footnote-ref-2)
2. POS restrictions were applied to certain procedure codes in order to exclude provide visits that occurred in nonambulatory settings. [↑](#footnote-ref-3)
3. POS restrictions were applied to certain procedure codes in order to exclude provide visits that occurred in nonambulatory settings. [↑](#footnote-ref-4)
